# RNA-sequencing analysis of *Trichophyton rubrum *transcriptome in response to sublethal doses of acriflavine

**DOI:** 10.1186/1471-2164-15-S7-S1

**Published:** 2014-10-27

**Authors:** Gabriela Felix Persinoti, Nalu Teixeira de Aguiar Peres, Tiago Rinaldi Jacob, Antonio Rossi, Ricardo Zorzetto Vêncio, Nilce Maria Martinez-Rossi

**Affiliations:** 1 Department of Genetics, Ribeirão Preto Medical School, University of São Paulo, Ribeirão Preto 14049-900, SP, Brazil; 2 Departamento de Computação e Matemática, Faculdade de Filosofia, Ciências e Letras de Ribeirão Preto, University of São Paulo, Ribeirão Preto 14049-900, SP, Brazil

## Abstract

**Background:**

The dermatophyte *Trichophyton rubrum *is an anthropophilic filamentous fungus that infects keratinized tissues and is the most common etiologic agent isolated in human dermatophytoses. The clinical treatment of these infections is challenging because only few antifungal drugs are commercially available. To understand the mode of action of cytotoxic drugs against fungi, we evaluated the time-dependent effects of acriflavine on *T. rubrum *transcriptome using high-throughput RNA-sequencing (RNA-seq) technology.

**Results:**

RNA-seq analysis generated approximately 200 million short reads that were mapped to the Broad Institute's Dermatophyte Comparative Database before differential gene expression analysis was performed. By employing a stringent cut-off threshold of −1.5 and 1.5 log_2_-fold changes in gene expression, a subset of 490 unique genes were found to be modulated in *T. rubrum *in response to acriflavine exposure. Among the selected genes, 69 genes were modulated at all exposure time points. Functional categorization indicated the putative involvement of these genes in various cellular processes such as oxidation-reduction reaction, transmembrane transport, and metal ion binding. Interestingly, genes putatively involved in the pathogenicity of dermatophytoses were down-regulated suggesting that this drug interferes with the virulence of *T. rubrum*. Moreover, we identified 159 novel putative transcripts in intergenic regions and two transcripts in intron regions of *T. rubrum *genome.

**Conclusion:**

The results provide insights into the molecular events underlying the stress responses of *T. rubrum *to acriflavine, revealing that this drug interfered with important molecular events involved in the establishment and maintenance of fungal infection in the host. In addition, the identification of novel transcripts will further enable the improvement of gene annotation and open reading frame prediction of *T. rubrum *and other dermatophyte genomes.

## Background

Acridine derivatives act on a myriad of biological targets, such as DNA-coiling enzymes (topoisomerases), telomerase/telomere, protein kinases, retrovirus integrases, hypoxia-selective environments, and play a role in the expression of genes coding for enzymes involved in mitochondrial respiratory-electron transport and iron transport [1]. The discovery of DNA intercalative properties of acridine led to the development of acridine derivatives for chemotherapy of different types of cancer [2,3]. Acriflavine (3,6-acridinediamine), an acridine derivative, is mutagenic and has been widely used to induce respiratory-deficient mutations in microorganisms such as the petite-phenotype yeasts [4,5]. A study that screened the effect of drugs in human colon cancer cell lines and in patient's tumor samples identified acriflavine as a promising drug for the treatment of colorectal cancer [6]. Acriflavine inhibits dimerization of HIF-1, a transcription factor that mediates adaptive responses to hypoxia and plays a critical role in cancer progression [7]. Acriflavine has been used as a topical antiseptic due to its antibiotic activity against fungi, bacteria, virus, and parasites [2,3]. It is also noteworthy that acriflavine is apparently oxidized *in vivo *to generate its active form [8].

The anthropophilic species *Trichophyton rubrum *is a filamentous fungus that infects keratinized tissues such as hair, nail, and skin [9]. It is the main cause of human dermatophytoses [10] and is responsible for invasive infections in immunocompromised patients. A correlation between cell adherence, keratinolytic activity, and pathogenesis has been proposed previously considering that during infection, the dermatophytes secrete a battery of endo- and exo-proteases that degrade these keratinized structures into oligopeptides and free amino acids, and use them as nutrients [11,12]. It is likely that proteases with optimal activity at both acidic and alkaline pH are important factors that determine the virulence of dermatophytes, and their regulation is a crucial determinant of the infection [13]. Therefore, the ability of *T. rubrum *to infect largely depends on its capability to alter its transcriptome in response to the natural host defenses.

The clinical treatment of fungal infection is prolonged, costly, and presents several challenges such as resistance to antifungal drugs and a limited number of cellular targets. Moreover, most commercially available antifungal drugs act on ergosterol, the main fungal-membrane sterol, or on the enzymes related to its biosynthesis. One drug that apparently does not act on ergosterol and has antifungal activity is acriflavine. Although acriflavine is not a commercial drug to treat dermatophytoses, it is worthwhile to evaluate its effects on the dermatophyte *T. rubrum *since it has a proven antifungal activity with a potential therapeutic effect. Therefore, acridine derivatives should be considered part of new therapeutic strategies that are being evaluated in several research programs.

We used RNA-sequencing (RNA-seq) analysis to evaluate *T. rubrum *transcriptome to identify the differentially expressed genes in response to acriflavine. The analysis indicated that a wide spectrum of genes are responsive to stress induced by acriflavine, including genes that determine the fungal virulence.

## Results

### Minimal inhibitory concentration of acriflavine

The susceptibility of *T. rubrum *to acriflavine was determined using the micro dilution assay, and the minimal inhibitory concentration (MIC) was found to be 2.5 μg/mL. The sub-inhibitory concentration of the drug used in the gene expression experiments corresponds to 70% of the MIC. This was adequate to cause a 15% reduction in fungal growth in the Sabouraud agar, as indicated by the colony diameter.

### Deep RNA sequencing of *T. rubrum*

To identify the changes in global transcriptome of *T. rubrum *after treatment with sub-inhibitory doses of acriflavine, we performed high-throughput RNA-seq. We sequenced more than 200 million reads [50 base pairs (bp) in length] corresponding to 4 barcode libraries. *T. rubrum *grown in Sabouraud medium served as control (0 h), and the treated specimen were exposed to acriflavine for 3, 12, and 24 h, combining three biological replicates each to increase reliability. Alignment was performed against the *T. rubrum *reference genome available at the Broad Institute using both the Bowtie [14] and TopHat [15] algorithms with different read lengths. In general, 60% of the total number of reads was aligned to *T. rubrum *reference genome (Additional File 1: Table S1). These data have been deposited in Gene Expression Omnibus (GEO) database under accession number GSE40425.

### Expression analysis

RNA-seq reads mapped to the *T. rubrum *reference genome available at the Broad Institute's Dermatophyte Comparative Database [16] were assembled into transcripts, and their relative abundances were estimated using Cufflinks algorithm [17]. Expression levels were measured in reads per kilo base of exon per million mapped reads (RPKM). The distribution of gene expression analyzed at each time point is illustrated in Additional file 2: Figure S1. The mean and median values at 0 h time point were 636.2 and 44.5, at 3 h were 484.1 and 51.2, at 12 h were 455.3 and 71.7, and at 24 h were 422.7 and 36.1. The differences observed between the mean and the median values are due to the intrinsic characteristics of the transcriptome, and reflect that a large number of genes are expressed at lower levels and a fewer number of genes are expressed at higher levels. When the genes with RPKM greater than 0 were considered, we identified 5,808 genes expressed at the 0 h time point, 7,464 at 3 h, 6,998 at 12 h, and 6,053 genes at 24 h, resulting in the identification of 66-85% of the 8,707 annotated genes expressed in *T. rubrum*. Among the most highly expressed genes, several encoded ribosomal proteins such as S23, L7, S12, S10-A, S20, S28, and L41.

**Figure 1:**
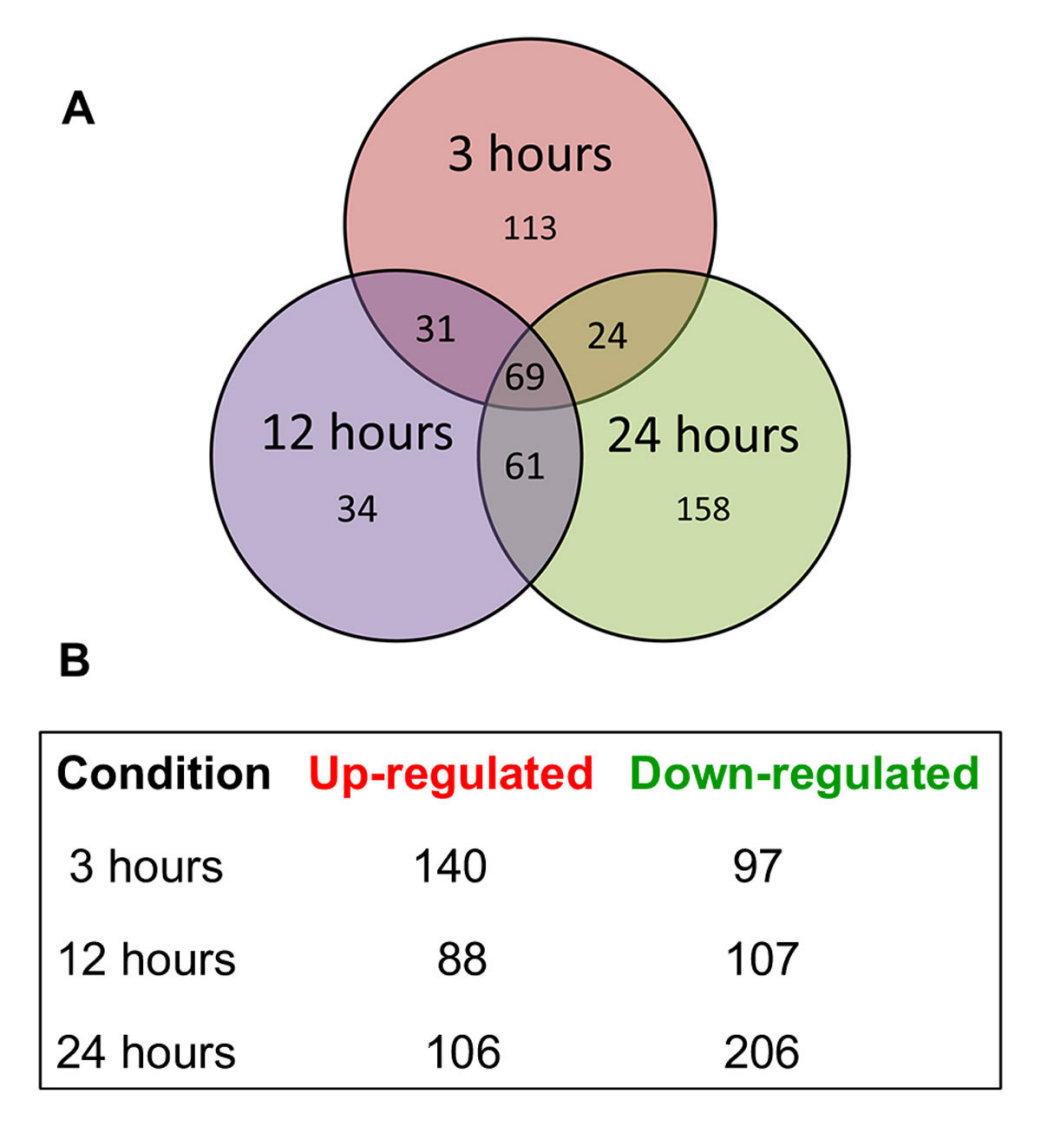
**Acriflavine-induced differential gene expression**. (A) Venn diagram illustrates the time-dependent differential expression of 490 genes of *Trichophyton rubrum*, after exposure to acriflavine for 3, 12, and 24 h, compared to the control (0 h). (B) Illustration of the number of up- or down-regulated genes in each experimental condition.

Differential gene expression was analyzed using the Cufflinks module Cuffdiff. Gene expression levels for different time points were analyzed in a pairwise comparison using the log_2 _ratio of each time point versus 0-h RPKM values. This analysis indicated that 3,153 genes were modulated in response to acriflavine, in which 2,734 were modulated at 3 h, 1,921 at 12 h, and 1,392 at 24 h, compared to the expression levels of the control. The volcano plot for each condition (Additional file 3: Figure S2) was analyzed by employing a stringent cut-off threshold of −1.5 and 1.5 log_2_-fold change in gene expression (i.e., more than 2.8-fold difference) and a stringent statistical significance threshold of p < 0.001. This analysis yielded a subset of 490 genes that were modulated in response to acriflavine (Additional file 4: Table S2) and were distributed as indicated in Figure 1. Among the 69 genes that were modulated at all three time-periods of exposure to acriflavine, 11 of them were up-regulated and 58 were down-regulated. The genes with the greatest up- and down-regulation in response to acriflavine are listed in Table 1. Genes involved in siderophore biosynthesis, detoxification, and nutrient transport are among the highly up-regulated genes. A total of 218 genes coding for hypothetical proteins were modulated in response to acriflavine exposure; of these, 33 were modulated in all three time points analyzed, being four of them consistently up-regulated while 29 were down-regulated (Table 2).

**Table 1:** The major up- and down-regulated genes after acriflavine exposure

ID	3 hours	12 hours	24 hours	Gene Product Name
TERG_00209	5.76	6.03	11.66	Hypothetical protein (tenascin C gene family)
TERG_00698	6.75	9.42	10.63	L-ornithine 5-monooxygenase
TERG_01685	8.26	9.98	9.21	glutathione transferase
TERG_00500	8.43	9.57	10.31	Hypothetical protein
TERG_02583	5.95	6.40	9.56	phosphate permease
TERG_04160	9.50	8.32	0	Hypothetical protein
TERG_03272	9.40	9.26	9.18	pre-mRNA branch site protein p14
TERG_07875	9.21	9.25	9.24	integral membrane protein
TERG_02897	9.08	9.30	9.24	methylene-fatty-acyl-phospholipid synthase
TERG_05361	7.20	9.03	8.84	cript family protein
TERG_05520	8.62	8.79	8.53	Hypothetical protein
TERG_02132	5.34	8.22	8.70	Hypothetical protein (Generic methytransferase gene family)
TERG_03274	8.44	7.16	7.88	ammonium transporter MeaA
TERG_05514	8.25	8.22	8.20	oligosaccharyltransferase subunit ribophorin II
TERG_01219	8.15	7.41	8.40	malate/L-lactate dehydrogenase
TERG_05137	8.11	5.30	0	microsomal dipeptidase
TERG_08522	7.86	6.88	7.61	pyruvate dehydrogenase E1 B-subunit
TERG_05478	6.96	6.06	6.28	chromatin remodeling complex subunit Arp5
TERG_01497	5.50	5.38	6.39	protein kinase subdomain-containing protein
TERG_05744	2.26	2.76	4.29	GTP-binding protein EsdC
TERG_07143	4.09	2.94	3.15	potassium/sodium efflux P-type ATPase
TERG_00697	1.24	2.79	3.51	non-ribosomal peptide synthetase
TERG_08353	0.66	0.98	2.97	cytochrome P450
TERG_05270	1.08	0.94	2.84	C2H2 finger domain-containing protein
TERG_03172	0.49	0.47	2.79	sodium/phosphate symporter
TERG_08201	1.20	1.55	2.63	serine protease
TERG_05854	-3.63	-3.98	-11.20	beta-lactamase
TERG_01454	-3.56	-2.81	-9.47	microsomal dipeptidase
TERG_08129	-3.48	-3.71	-4.64	amino acid permease
TERG_01401	-3.78	-2.15	-4.06	high affinity copper transporter
TERG_07734	-2.47	-4.04	-3.93	O-methyltransferase
TERG_01609	-0.26	-2.80	-3.66	Na/K ATPase alpha 1 subunit
TERG_02400	-1.93	-3.01	-3.62	amino acid permease
TERG_03352	-2.23	-2.74	-3.48	tyrosine decarboxylase
TERG_04146	-2.40	-2.77	-3.47	FAD binding domain-containing protein
TERG_01346	-1.36	-2.83	-3.40	lipase/serine esterase
TERG_06701	-2.68	-3.38	-3.19	gamma-glutamyltranspeptidase
TERG_04238	-1.28	-1.47	-3.38	cation-transporting ATPase
TERG_00066	-0.67	-2.58	-3.32	4-hydroxyphenylpyruvate dioxygenase
TERG_07214	-1.47	-1.78	-3.29	DlpA domain-containing protein
TERG_00227	-1.72	-3.05	-3.25	glutathione S-transferase
TERG_05332	-1.23	-1.61	-3.12	mitochondrial carrier protein
TERG_00546	-1.08	-0.78	-3.04	Glutaredoxin
TERG_06938	-0.59	-1.95	-3.01	zinc metallopeptidase
TERG_05409	-1.84	-2.94	-2.98	FAD dependent oxidoreductase

**Table 2:** Cellular transport-related genes modulated in response to acriflavine exposure

Genes related to transport				
**ID**	**3 hours**	**12 hours**	**24 hours**	**Gene Product Name**

TERG_07801	-0.69	2.60	3.13	ABC multidrug transporter Mdr4
TERG_02369	1.26	1.81	1.75	MFS transporter
TERG_01443	1.80	1.46	1.52	ABC multidrug transporter
TERG_04952	2.14	1.41	0.97	ABC transporter
TERG_01623	0.43	1.10	1.72	MFS transporter
TERG_00008	-0.39	-0.36	-1.68	MFS phospholipid transporter
TERG_02283	-0.34	-0.67	-1.61	MFS transporter
TERG_07027	-1.18	-0.78	-2.08	MFS drug transporter
TERG_03933	-0.93	-1.37	-1.73	ABC metal ion transporter
TERG_08336	-1.19	-1.42	-1.59	MFS multidrug transporter
TERG_00820	-1.06	-1.49	-2.30	MFS multidrug resistance transporter
TERG_08130	-1.29	-1.70	-1.77	ABC transporter
TERG_08613	-1.09	-1.70	-2.14	Multidrug resistance protein Mdr2
TERG_00955	-1.66	-1.79	-2.29	ABC efflux transporter
TERG_05153	-1.48	-1.89	-2.37	MFS transporter
TERG_00402	-1.60	-2.10	-2.25	Multidrug resistance protein
TERG_06161	-2.42	-2.26	-2.66	Multidrug resistance protein

**Genes related to iron and zinc transport**				

**ID**	**3 hours**	**12 hours**	**24 hours**	**Gene Product Name**

TERG_03174	0.47	3.69	4.97	Siderochrome-iron transporter Sit1
TERG_06788	5.01	0.65	9.66	Zinc/iron transporter
TERG_08090	0.34	1.24	1.73	Plasma membrane iron permease
TERG_08619	-2.76	-0.95	-1.72	Siderophore iron transporter
TERG_08620	-2.25	-0.93	-2.61	Siderophore iron transporter mirB
TERG_05855	5.02	7.53	9.73	ZIP zinc transporter

### Functional categorization of differentially expressed genes

**Figure 2:**
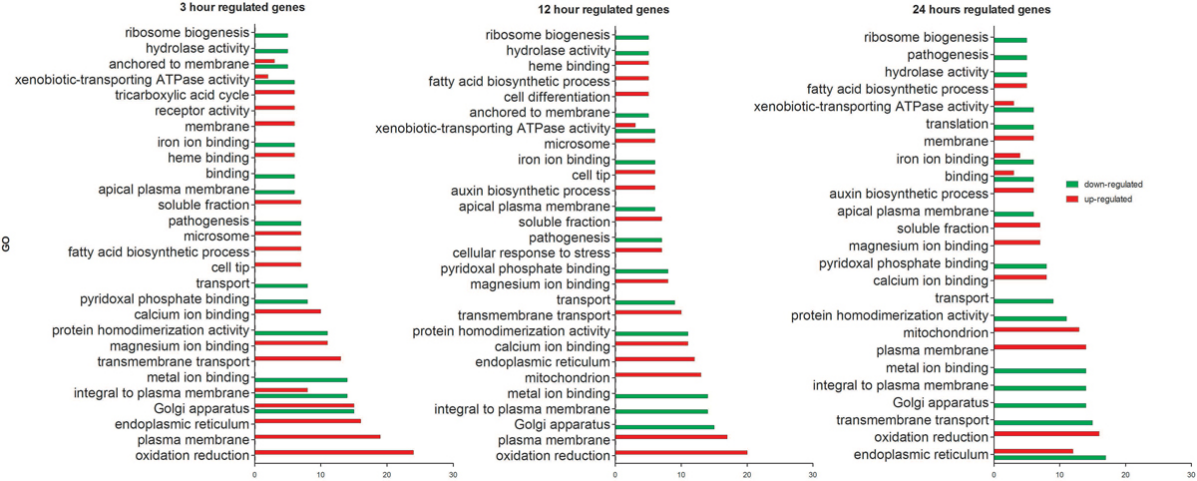
**Gene ontology-based functional categorization of the differentially expressed genes**. The significantly enriched (p < 0.05) GO categories are represented, with the red and green bars indicating the number of up- and down-regulated genes, respectively.

To enhance our understanding of the molecular mechanism by which *T. rubrum *senses and responds to stress caused by acriflavine, we examined the functional distribution of the modulated genes using the Blast2GO [18] and BayGO [19] tools. Genes with 1.5 log_2_-fold change were analyzed and their gene ontology (GO) functions are presented in Figure 2. This analysis indicated that the genes modulated by acriflavine were involved in several cellular processes such as oxidation-reduction reactions, transmembrane transport, metal-ion binding, fatty acid biosynthesis, and pathogenicity. The over represented GO categories, in response to acriflavine, included several genes related to cellular transport, which are important for stress responses, such as genes encoding the ATP-binding cassette (ABC), the major facilitator super-family (MSF) of transporters, and genes related to metal ion transport (Table 2). Most of the genes encoding for cellular transporters were down-regulated, whereas the Mdr4 transporter gene was up-regulated in response to the drug. A group of genes related to the endoplasmic reticulum was up-regulated at all the analyzed time points, while another sub-group was down-regulated only at 24 h (Figure. 2). Furthermore, genes associated with the pathogenesis of dermatophytoses, particularly those encoding proteases, were down-regulated at all three time points (Figure 2).

### Quantitative real-time PCR

**Figure 3:**
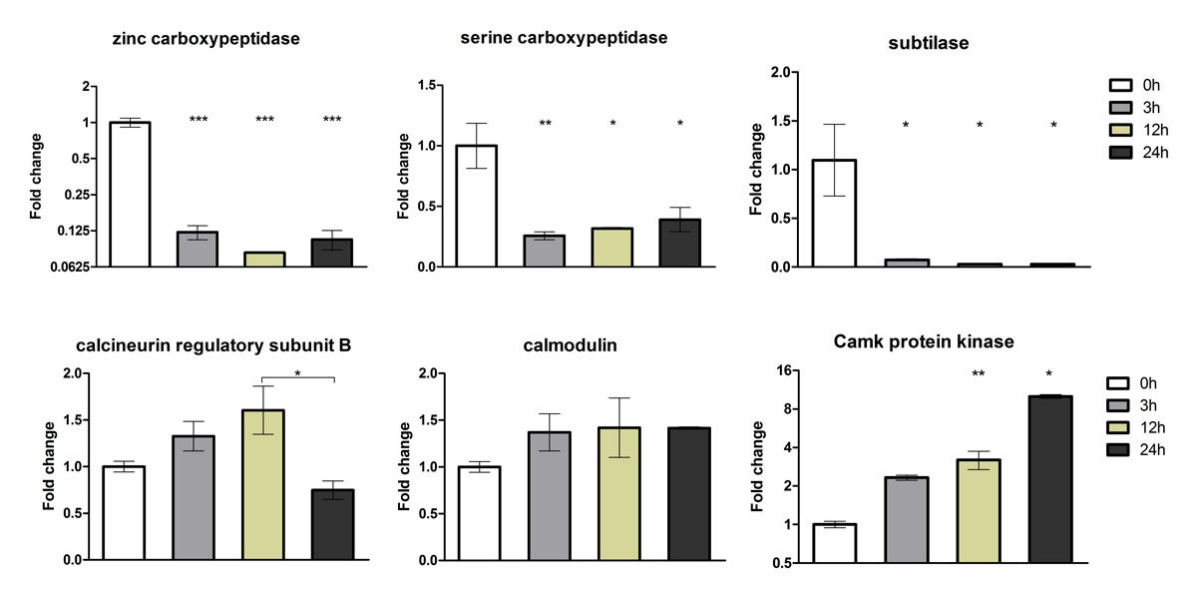
**Validation of differentially expressed genes using quantitative real-time PCR (qRT-PCR)**. Six genes that were modulated in response to acriflavine were amplified using the cDNA obtained from the mycelia of *T. rubrum *exposed to acriflavine for 3, 12, and 24 h. The gene expression levels are represented by the fold changes at each time point relative to the control (0 h). The genes are listed in Table 3. The results of the qRT-PCR assay, from two independent experiments, are expressed as mean ± standard deviation (S.D). Statistical significance was determined using Bonferroni's *ad hoc *test and indicated by asterisks: * indicates p < 0.05; ** indicates p < 0.01; *** indicates p < 0.001.

**Figure 4:**
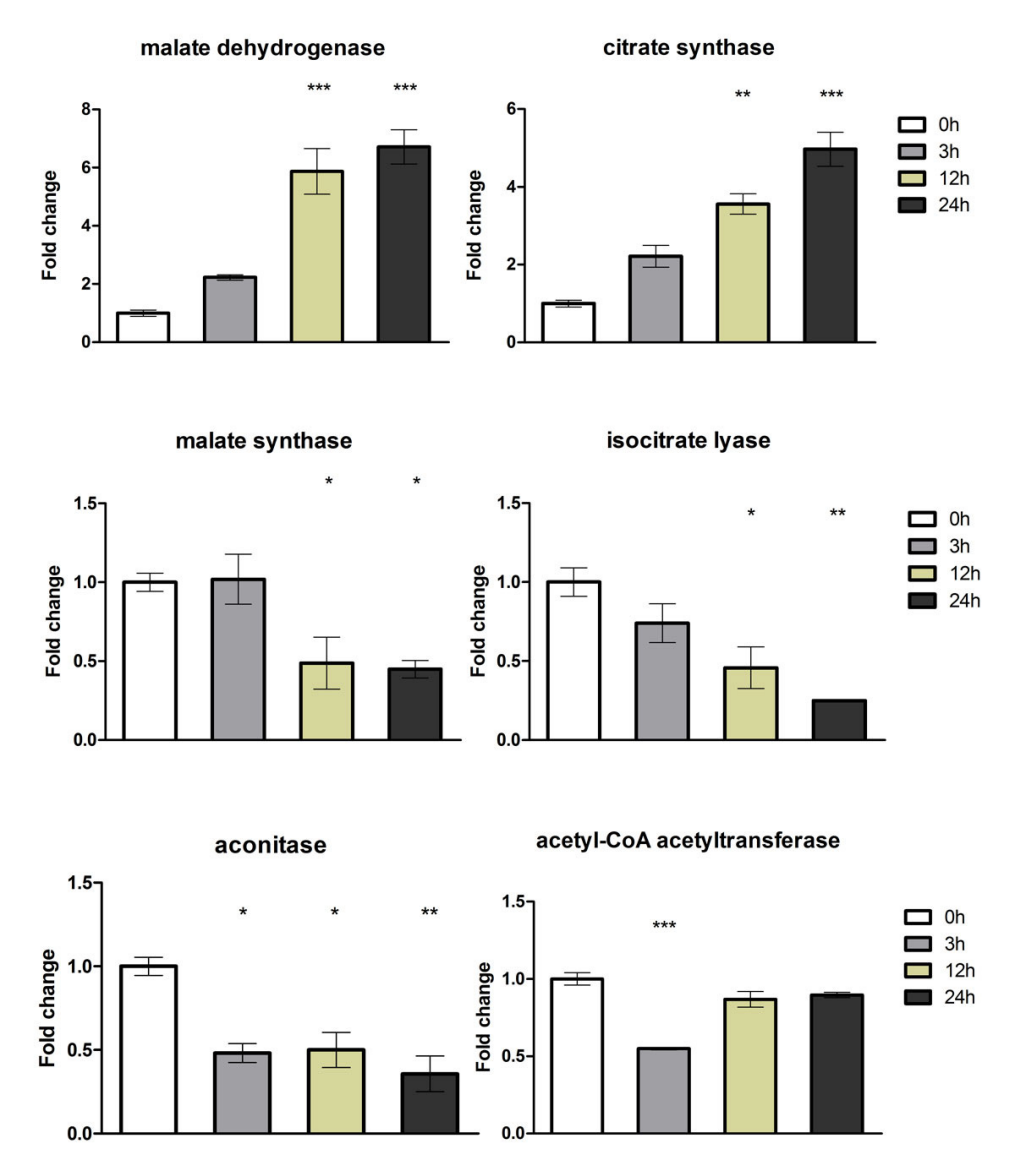
**Validation of differentially expressed genes using quantitative real-time PCR (qRT-PCR)**. Six genes that were modulated in response to acriflavine were amplified using the cDNA obtained from the mycelia of *T. rubrum *exposed to acriflavine for 3, 12, and 24 h. The gene expression levels are represented by the fold changes at each time point relative to the control (0 h). The genes are listed in Table 3. The results of the qRT-PCR assay, from two independent experiments, are expressed as mean ± standard deviation (S.D). Statistical significance was determined using Bonferroni's *ad hoc *test and indicated by asterisks: * indicates p < 0.05; ** indicates p < 0.01; *** indicates p < 0.001.

**Figure 5:**
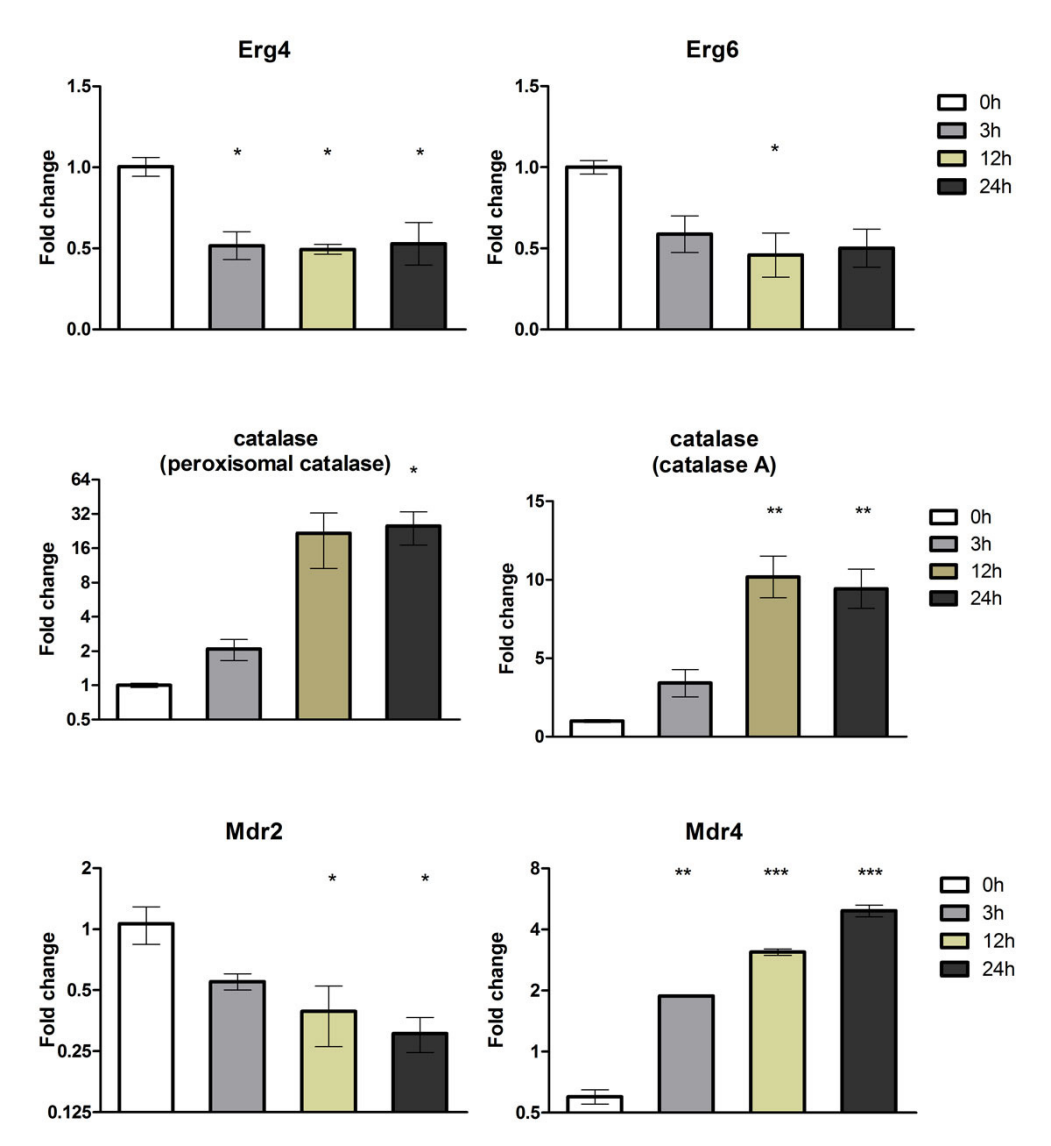
**Validation of differentially expressed genes using quantitative real-time PCR (qRT-PCR)**. Six genes that were modulated in response to acriflavine were amplified using the cDNA obtained from the mycelia of *T. rubrum *exposed to acriflavine for 3, 12, and 24 h. The gene expression levels are represented by the fold changes at each time point relative to the control (0 h). The genes are listed in Table 3. The results of the qRT-PCR assay, from two independent experiments, are expressed as mean ± standard deviation (S.D). Statistical significance was determined using Bonferroni's *ad hoc *test and indicated by asterisks: * indicates p < 0.05; ** indicates p < 0.01; *** indicates p < 0.001.

The RNA-seq results were validated using quantitative real-time PCR (qPCR) for 18 selected genes using independent RNA samples, i.e., biological replicates (Figures 3, Figure 4 and Figure 5). It is important to note that our conclusions are based on the use of independent RNAs for validation. The genes evaluated by qPCR were chosen considering their involvement in pathogenicity, glyoxylate cycle, ergosterol biosynthesis pathway, cell detoxification, and transport. The results of both experiments were compared with respect to the log_2 _ratio between each time point analyzed and the reference sample at 0 h (Table 3). The correlation between the SOLiD RNA-seq and qPCR results, obtained from biological replicates, was strong and was statistically significant (Pearson correlation, r = 0.85, p < 0.001).

**Table 3:** Comparison of the gene expression levels assayed by RNA sequencing and qPCR

ID	Gene Product Name	3 h		12 h		24 h	
		**RNA-seq**	**qPCR**	**RNA-seq**	**qPCR**	**RNA-seq**	**qPCR**

TERG_02214	Zinc carboxypeptidase	-2.59	-3.11	-3.51	-3.69	-4.20	-3.36
TERG_07861	Subtilase	-2.54	-3.91	-3.61	-5.30	-4.81	-5.31
TERG_08557	Serine carboxypeptidase	-1.54	-1.99	-0.92	-1.69	-2.14	-1.42
TERG_06573	Calcineurin regulatory subunit B	-0.78	0.41	-1.28	0.68	-2.25	-0.42
TERG_06392	Calmodulin	0.97	0.45	0.78	0.51	1.02	0.50
TERG_02198	CamK protein kinase	0.29	1.22	0.74	1.68	1.68	3.33
TERG_01281	Malate synthase	-0.67	0.03	-1.42	-1.06	-1.83	-1.16
TERG_03762	Malate dehydrogenase	1.15	1.11	1.14	2.45	2.21	2.64
TERG_01871	Acetyl-coa acetyltransferase	1.71	-0.86	1.28	-0.20	1.43	-0.16
TERG_04125	Citrate synthase	1.71	0.62	1.73	1.68	2.40	2.15
TERG_00825	Isocitrate lyase	-1.22	-0.64	-2.18	-1.50	-2.69	-2.45
TERG_01076	Aconitase	0.73	-0.95	-0.31	-0.86	-0.92	-1.31
TERG_02979	Delta(24(24(1)))-sterol reductase (Erg4)	-1.06	-0.80	-1.39	-0.86	-1.91	-0.70
TERG_03102	Sterol 24-C-methyltransferase (Erg6)	-1.31	-0.31	-0.82	-0.68	-1.54	-0.53
TERG_01252	Catalase (catalase A)	1.70	1.77	2.45	3.35	2.80	3.24
TERG_06053	Catalase (peroxisomal catalase)	6.09	1.01	7.13	4.26	6.49	4.43
TERG_08613	Multidrug resistance protein Mdr2	-1.09	-0.85	-1.70	-1.34	-2.14	-1.70
TERG_07801	ABC multidrug transporter Mdr4	-0.69	0.91	2.6	1.63	3.13	2.30

### Isocitrate lyase (ICL) activity

ICL specific activity, measured in cultures of *T. rubrum *exposed to acriflavine for 0h, 3h, 12h and 24h (the same culture conditions used for the RNA-seq assay) was 0.101, 0.064, 0.139 and 0.095 U/mg protein, respectively. These results did not show statistical significance when compared treated mycelia to the non-treated.

### Identification of novel transcripts

**Figure 6:**
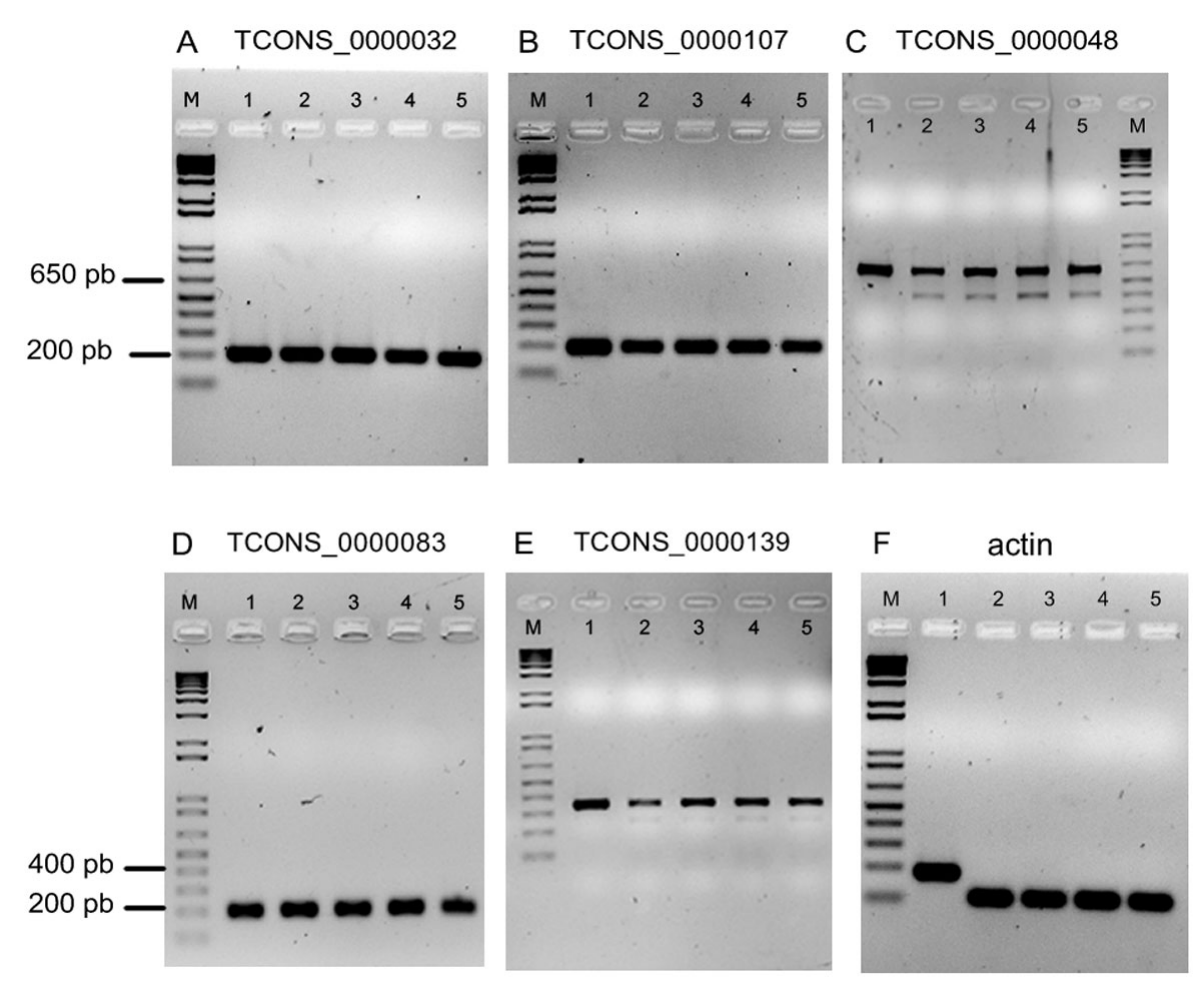
**RT-PCR assay of novel transcribed regions**. (A-E) The nucleotide sequence of each transcript in *Trichophyton rubrum *genome is presented in Additional file 5: Table S3. The lanes labeled M, 1, 2, 3, 4 and 5 represents the 1 kb plus molecular weight ladder, the *T. rubrum *genomic DNA, *T. rubrum *cDNA before and after acriflavine exposure for 0, 3, 12, and 24 h, respectively. (F) The actin gene was used as a control. The primers used to amplify both the actin gene and the transcripts were designed to flank an intron of the gene.

For identifying novel transcript fragments, we used Cufflinks [17] without any previous gene annotations. This analysis revealed 161 novel transcripts when the combined alignment of the four experimental conditions was analyzed. Comparing the structure of these transcribed genomic regions with previous annotations, we identified 159 novel transcripts of intergenic regions and two transcripts of intronic regions in the *T. rubrum *genome (Additional file 5: Table S3). The putative novel transcripts are relatively shorter than *T. rubrum *annotated genes, with a mean and median length of 1,075 bp and 1,008 bp, respectively, whereas the annotated genes have a mean length of 1,598 and a median of 1,318 bp. To support the validity of these novel transcribed intergenic regions, five of them were checked by RT-PCR, and this analysis confirmed their transcription (Figure 6, Additional file 6: Table S4).

## Discussion

RNA-seq analysis is a highly valuable resource in the field of molecular genetics as it facilitates a better understanding of the complex biological systems. We used this global transcriptome analysis to identify differentially expressed genes when *T. rubrum *was challenged with sublethal doses of acriflavine. At least 37 million quality-filtered reads for each sample was generated, with an alignment rate of approximately 60% for each library. This relatively low alignment rate is an intrinsic characteristic of SOLiD 4 System sequencing [20]. We observed that 66-85% of the *T. rubrum *annotated genes were expressed in at least one of the experimental conditions. GO-based functional categorization of a subset of 490 unique genes indicated that the genes with more than 2.8-fold difference in their expression levels in response to acriflavine exposure were associated with several critical cellular functions. The most highly represented GO categories included transmembrane transport, metal ion-binding, fatty acid biosynthesis, oxidation-reduction reactions, and pathogenicity, indicating that this drug has a broad spectrum of cellular effects. Although some modulated genes may be related to fungal growth during exposure to acriflavine over 24 hours, we used in the derepression assays the mycelium obtained after the conidia were cultured for 96 hours (stationary growth phase). Moreover, the modulation of genes involved in transmembrane transport, pathogenicity, glyoxylate cycle and oxidation-reduction, revealed in this work, is typically cellular adaptive response to stress.

### Genes involved in transmembrane transport

ABC and MFS transporters are two major classes of proteins involved in drug resistance, with a large range of substrates such as ions, amino acids, peptides, sugars, secondary metabolites, and drugs. These proteins are highly conserved among bacteria, fungi, plants, and animals, and play important roles in the efflux of toxic components and metabolites from the cell. Furthermore, these proteins are involved in a large variety of molecular events such as protein secretion, nutrient uptake, and pathogenesis [11,21]. Most of these genes were down-regulated (Table 2), whereas the Mdr4 (Figure 5) was up-regulated in response to acriflavine, suggesting that Mdr4 may have a higher affinity to the drug, and is possibly involved with the efflux of acriflavine from the cell in order to enhance the cell viability. In contrast, the Mdr2 transporter (*Tru*MDR2 gene), a multidrug transporter of the ABC family, which is involved in *T. rubrum *resistance to some antifungal drugs, was down-regulated (Table 2, Figure 5). In previous studies, expression of this gene was analyzed in response to several cytotoxic agents and it was found to be up-regulated after exposure to acriflavine for 15 min [22,23]. We showed down-regulation of *Tru*MDR2 gene after 3 h, indicating that it could have a functional role during the early response to acriflavine. Moreover, deletion of *Tru*MDR2 gene did not alter the susceptibility of *T. rubrum *to acriflavine, suggesting a functional overlap among different transporters [23]. The decreased expression of this gene and other transporter genes during treatment with acriflavine suggests that the drug interferes with crucial processes that enable cell detoxification and growth. Moreover, the *Tru*MDR2 was also shown to be important for *T. rubrum *growth in keratin and in human nails [21]. Differential expression of the genes involved in the regulation of several transporters would be important for maintaining cell viability during both stressful and physiological conditions.

In addition to ABC and MSF transporters we also identified differentially expressed genes related to metal ion transport, particularly iron transporters. Iron is an important factor for the growth of microorganisms and fungal infections [24]. It is indispensable in different cellular processes such as respiration, the tricarboxylic acid cycle, detoxification of oxidative stress, and the synthesis of amino acids, deoxyribonucleotides, lipids, and sterols [25]. In response to iron depletion, fungi secrete siderophores, i.e., iron chelator molecules with low molecular weight, which are considered virulence factors of fungal species such as *Aspergillus fumigatus *[26]. Acriflavine exposure-induced overexpression of an MFS, similar to a siderophore iron transporter, has been previously reported in *T. rubrum *[1]. Two of the genes among the highly up-regulated are required for the biosynthesis of siderophore: L-ornithine 5-monooxygenase and non-ribosomal peptide synthase. This suggests that *T. rubrum *requires iron to overcome the toxic effects of sub inhibitory concentration of acriflavine. This may be caused by the lack of iron due to the low expression of some transporter genes, including siderophore iron transporter genes under similar experimental conditions (Table 2). Siderophore-bound iron can be recovered in cells by specific transporters capable of internalizing the siderophore-iron complex[24]. The broad-spectrum analysis of the gene expression performed here indicated that, similar to the genes coding for transporters, the molecules involved in iron and zinc transport also appear to exhibit different modulations and roles in the cell (Table 2). Up-regulation of some transporters and down-regulation of others in response to acriflavine suggest that a balanced expression of different transporters would be important for fungal adaptation to stress caused by the drug, which may be essential for maintaining cell viability.

### Genes involved in pathogenicity

Modulation of *T. rubrum *genes coding for known virulence factors in dermatophytes and other fungal species in response to acriflavine was also revealed here. The most studied virulence factor of dermatophytes includes enzymes that are released during the infection process allowing the utilization of the host tissue nutrients, enabling fungal invasion and dissemination [27]. It has been proposed that during the infection process, each dermatophyte has a specific system for the regulation of proteases and other virulence factors according to the specificity of each host [28]. Thus, for successful hyphal development in the host, these fungi express several proteases and peptidases that are secreted into the host microenvironment, allowing the cleavage and uptake of proteins and amino acids. Interestingly, most genes categorized using GO analysis as being involved in the pathogenicity were down-regulated in response to acriflavine, indicating that this drug interferes with genes that facilitate the establishment and maintenance of infection.

The calcium-signaling pathway, which is mediated by calmodulin and calcineurin, is related to the virulence and regulation of stress responses in microorganisms. This signal transduction cascade is important for diverse cellular processes in eukaryotes, including the maintenance of cellular homeostasis. Calmodulin is a calcium-binding protein, and calcineurin is a serine-threonine-specific phosphatase composed of catalytic subunit A, which contains the calmodulin-binding domain, and regulatory subunit B. In *Aspergillus fumigatus*, calcineurin is associated with conidiation, fungal growth, and pathogenicity [29]. Calmodulin responds to stress by activating calcineurin and calcium-calmodulin-dependent protein kinases [30]. In pathogenic fungi such as *Candida albicans *and *Cryptococcus neoformans*, calcineurin plays a critical role in determining their virulence [31]. Our results indicated a decrease in the expression of the calcineurin gene in response to acriflavine exposure at 24 h, whereas calmodulin was expressed at basal levels. In contrast, the gene coding for a calcium-calmodulin-dependent protein kinase (CamK protein kinase) was up-regulated mainly after exposure of *T. rubrum *to acriflavine for 24 h (Figure 3), suggesting that the differential modulation of this signaling pathway is important for maintaining cell survival under stressful conditions in dermatophytes.

### Genes involved in glyoxylate cycle

The glyoxylate cycle present in microorganisms and plants allows the metabolism of 2-carbon substrates, such as ethanol and acetate, to produce glucose when these substrates are the only carbon sources available [32]. The enzymes malate synthase and isocitrate lyase, which are exclusive components of this metabolic route, have been proposed as virulence factors in the human pathogenic fungus *C. albicans *[33] and in the phytopathogen *Magnaporthe grisea *[34]. In human pathogens such as *Mycobacterium tuberculosis *and the dimorphic fungus *C. albicans*, the glyoxylate cycle is induced during phagocytosis by macrophages and is required for fungal virulence. It has been proposed that this pathway is induced in the phagosome in response to nutrient starvation, allowing nutrient uptake and survival, thus making them important as virulence factors. Moreover, *C. albicans *mutants lacking the gene encoding isocitrate lyase has decreased virulence in a murine model [32]. However, in another study, the deletion of the gene encoding malate synthase in *Arthroderma benhamiae*, a dermatophyte that infects animals, did not reduce its capacity to infect guinea pigs, although a reduced ability to grow was observed with lipids as the carbon source [35]. In *T. rubrum*, both malate synthase and isocitrate lyase are up-regulated in media supplemented with proteins [36]. However, the precise role of the glyoxylate cycle has not yet been elucidated in dermatophyte development and virulence. Our results clearly suggest the accumulation of transcripts of key enzymes comprising one part of this cycle (malate dehydrogenase and citrate synthase), while the other part was repressed (aconitase, isocitrate lyase, and malate synthase), indicating a compensatory response against the repression of genes coding for enzymes of the glyoxylate cycle by acriflavine. However, this balance could be much more complex since the transcription level of the ICL gene did not directly correlates with the quantity of the enzyme activity present in *T. rubrum *mycelia. Indeed, there are several examples in the literature where this correlation does not exist due to post-transcriptional and post-translational events [37,38].

### Genes involved in ergosterol biosynthesis pathway

Ergosterol, a cholesterol analogue, is the major sterol of the fungal plasma membrane contributing to a variety of cellular functions such as fluidity and integrity. A reasonable number of antifungal agents are currently available in the pharmaceutical market, together with some derivatives of these drugs that have become less toxic, with enhanced potencies and improved pharmacokinetics. However, their cellular targets are limited because the common antifungal drugs are directed against the ergosterol biosynthetic pathway, with few exceptions (e.g., griseofulvin, flucytosine, caspofungin, and ciclopiroxolamine) [39]. The proper functioning of many membrane-bound enzymes, including chitin synthase, which is crucial for cell growth and division, is also dependent on ergosterol for the maintenance of the membrane's native conformation [40,41]. However, the significant incidence of fungal infections on the growing population of immunocompromised patients and the emerging resistance to existing drugs emphasize the importance of molecular studies concerning antifungal resistance, which in turn may be valuable in the search for new targets and in the improvement of the existing antifungals. In our analysis, acriflavine was found to interfere with the expression of the genes coding for components of the ergosterol biosynthetic pathway, Delta(24(24(1)))-sterol reductase (*erg4*) and sterol 24-C-methyltransferase (*erg6*). In *T. rubrum*, down-regulation of the *erg4 *gene in response to terbinafine and ketoconazole, which are drugs that interfere with squalene epoxidase and cytochrome P450 14α-lanosterol demethylase, respectively, has been reported. Both of these enzymes belong to the ergosterol biosynthesis pathway [42,43]. In contrast, *erg6 *has been found to be up-regulated in response to ketoconazole and itraconazole [43,44]. However, in our study, both *erg4 *and *erg6 *were down-regulated in response to acriflavine, suggesting the existence of a new potential molecular mechanism for acriflavine action, including interference with biosynthesis of membrane components, which may account for the reduction in fungal growth during drug exposure.

### Genes involved in oxidation-reduction

Catalases are enzymes responsible for the degradation of hydrogen peroxide (H_2_O_2_) and protection of the cell against oxidative stress and reactive oxygen species (ROS) accumulation [45,46]. Acriflavine inhibits catalase activity *in vitro *in a competitive manner, which correlates with the conformational alterations of the enzyme structure caused by the inhibitory effect as previously observed by fluorescence spectroscopy [47]. Moreover, acriflavine induces both apoptosis and necrosis in the yeast *Candida utilis *[48]. Apoptosis is a programmed cell death mechanism associated with cellular homeostasis. Characteristic changes in apoptotic cells include the accumulation of ROS, breaks in DNA, and activation of caspases [49]. The H_2_O_2 _produced during aerobic metabolism or in response to different types of stress may generate ROS. Our results revealed the modulation of two genes encoding catalases in response to acriflavine. Therefore, up-regulation of catalases may be the result of a compensatory mechanism for increasing catalase activity, which is inhibited by acriflavine in an attempt to protect the cell against acriflavine-induced apoptotic effects.

### Identification of novel transcripts

An interesting feature of RNA-seq technology is the possibility to identify novel transcripts. In this analysis, we identified 159 novel transcripts of intergenic regions and two transcripts of intronic regions in the *T. rubrum *genome (Additional file 5: Table S3). These transcripts were also compared to six other dermatophyte genomes [16], and all transcripts were identified in at least two of these dermatophytes. To further characterize these transcribed genomic regions we performed alignments with the GenBank NR (NCBI) and Rfam (Wellcome Trust Sanger Institute) [50]. Among the transcripts analyzed, 66 were similar to the genes already described in other organisms, although more than 80% of them are described as hypothetical proteins. In addition, several non-coding RNAs, that include 12 tRNAs and 9 small nucleolar RNAs (snoRNAs), were identified, which are involved in the chemical modifications of other RNAs such as ribosomal RNAs. However, most of the transcribed regions identified here are still uncharacterized, suggesting that these transcripts may constitute novel genes of *T. rubrum*. Therefore, further studies are required to elucidate their functions. Interestingly, two of the transcribed regions selected for RT-PCR assays (Figure 6C, Figure 6E) presented two putative alternative isoforms, as indicated by two different amplification products in RT-PCR experiments. These two transcribed genomic regions were further cloned and re-sequenced to confirm the alternative spliced isoforms. Our results reinforce the requirement of further research using the RNA-seq technology to improve the gene annotation for *T. rubrum *and other dermatophyte genomes.

## Conclusion

The large-scale sequencing of the *T. rubrum *transcriptome showed differential modulation of genes involved in various cellular processes in response to exposure of this dermatophyte to the cytotoxic drug acriflavine. Genes down-regulated in response to acriflavine included those encoding proteases, which are known virulence factors in dermatophytes. This suggests that acriflavine interferes with important factors involved in the establishment and maintenance of fungal infection in the host.

In addition, we identified novel transcribed genomic regions in this study that will further enable the improvement of gene annotation and open reading frame prediction of *T. rubrum *and other dermatophyte genomes.

## Materials and methods

### Biological samples

*T. rubrum *strain CBS118892 (*Centraalbureau voor Schimmelcultures*, Netherlands) fully produces conidia after growth at 28°C in malt extract agar for 15 d. Approximately, 1 × 10^6 ^conidia was obtained as previously described [51] and inoculated into 100 mL of Sabouraud media and incubated at 28°C for 96 h under agitation. Next, the mycelia were aseptically transferred to RPMI 1640 media (Gibco, USA) containing 1.75 µg/mL of acriflavine (Sigma, USA), which corresponds to 70% of its MIC. After 3, 12, and 24 h of incubation at 28°C under agitation, the resultant mycelia were collected and stored at −80°C until RNA isolation. Several biological replicates were grown in order to harvest RNA for sequencing.

### Determination of MIC

MIC is defined as the lowest drug concentration that inhibits macroscopic fungal growth. *T. rubrum *susceptibility to acriflavine was evaluated by assessing MIC using the microdilution approach (M38-A) proposed by the Clinical and Laboratory Standards Institute (CLSI). All the assays were carried out in triplicate at 28°C for 5 d. The concentrations assayed were serial dilutions ranging from 0.039 to 10 μg/mL of acriflavine diluted in water.

### RNA extraction

Total RNA was isolated from approximately 100 mg of mycelia using the Illustra RNAspin Mini Isolation Kit (GE, USA). RNA concentrations were determined using a NanoDrop ND-1000 spectrophotometer, and RNA quality was verified using both agarose electrophoresis and the Agilent 2100 Bioanalyzer (Agilent, USA). High-quality mRNA was obtained by removing large and small ribosomal RNA from the samples with the RiboMinus Kit (Invitrogen, USA).

### Library construction and SOLiD sequencing

To improve reliability, total RNA from three biological replicates at each time point (0, 3, 12, and 24 h) was pooled for the preparation of the next-generation sequencing libraries to decrease biological noise. RNA was fragmented using the SOLiD 4 Total RNA Seq Kit (Applied Biosystems, USA), and was reverse transcribed using ArrayScript Reverse Transcriptase (Ambion, USA). The cDNA was synthesized and purified with the Qiagen MinElute PCR Purification Kit (Qiagen, USA) and was run on a Novex 6% TBE-urea gel (Invitrogen, USA) for size selection. The cDNA was excised from the gel at a size range of 150-250 bp, and in-gel PCR reactions were performed to obtain adequate material for subsequent emulsion PCR (ePCR). During this PCR reaction, each library was barcoded using PCR primers with different barcodes to allow for multiplex sequencing. The ePCR and emulsion break were performed following the Applied Biosystems SOLiD 4 System Templated Bead Preparation Guide. The amplified beads were first run on a workflow analysis slide to determine the quality and quantity of the beads, which was followed by a single sequencing run, performed according to the Applied Biosystems SOLiD 4 System Instrument Operation Guide. Both library construction and sequencing were performed at Cofactor Genomics (USA).

### Data analysis

The SOLiD 4 System was used to generate color-space encoded reads that were 50 bp long. Reads were first assigned to each sample by matching the decoded barcode used for each library. Next, reads were quality-filtered using a method developed specifically for SOLiD reads [52]. Alignment with the *T. rubrum *genome version 2 available at the Broad Institute's Dermatophyte Comparative Database was performed using both the TopHat [15] and Bowtie algorithms [14], which index the reference genome in color-space to perform the alignment and prevent the scaling-up of mismatches. Gene expression profiling analysis was based on the number of reads mapped to the *T. rubrum *transcripts, and relative abundance was expressed in RPKM, calculated using RNA-seq fragment counts. The aligned reads were processed by the Cufflinks [17] and Cuffdiff software for assembling the transcripts, estimating transcript abundance, and testing for differential expression between control and acriflavine-treated samples. Cufflinks can handle multi-mapped reads by first calculating the initial abundance estimated for all transcripts and then re-estimating the abundances of transcripts using a probabilistic assignment of multi-mapped reads based on the initial estimation. Genes with p-values less than 0.001 were considered to be differentially expressed, and those presenting log_2_-fold change greater than 1.5 or lower than −1.5 (i.e, at least 2.8-fold difference) were functionally categorized according to the GO using the Blast2GO [18] algorithm. Enrichment analysis was performed using the BayGO algorithm [19]. Mapping results were visualized using both the Gaggle Genome Browser [53] and Genome View [54]. Pileup coverage files were uploaded to the Gaggle Genome Browser to inspect the read coverage achieved.

### The qPCR analysis

The expression of selected genes was quantified by performing qPCR with the StepOnePlus Real-Time PCR System (Applied Biosystems, USA). Two independent samples from each time point analyzed were used for qPCR analysis, and the reactions were performed in triplicate. Specific primer pairs were designed using the Primer3 software, and their specificity was confirmed by BLAST searches against the *T. rubrum *genome database. The sequences of the primers are listed in Additional file 7: Table S5. The qPCR experiments were performed in a 12.5-µL reaction containing the SYBR Green PCR Master Mix (Applied Biosystems), 50 ng of cDNA, and 1 µL of each primer. The PCR protocol included an initial denaturation at 95°C for 10 min, followed by 40 cycles of 95°C for 15 s and 60°C for 1 min. A dissociation curve was generated at the end of each PCR cycle to verify the amplification of a single product. The 2^-ΔΔCt ^relative expression quantification method was used to calculate the fold change in each gene, using the *rpb2 *gene as a reliable reference control [55]. Analysis was performed using the StepOne Software v2.2. The reference sample at 0 h was used to calculate relative gene expression levels. Statistical analysis was performed using one-way ANOVA followed by the Bonferroni's *ad hoc *test using Graph Pad Prism v 5.1 Software.

### Protein extraction and measure of isocitrate lyase (ICL) activity

*T. rubrum *was grown as described for RNA extraction, frozen, and total protein was extracted by grounding the mycelia to a fine powder, resuspended in 1mL of Tris-HCl buffer (50 mM Tris-HCl, 2 mM MgCl_2_, 2 mM DTT, pH 8.0), mixed by vortex and centrifuged for 30 min at 4°C at 1270 × g. The pellet was discarded and the supernatant was used for protein quantification using the Bradford Reagent, at 595 nm. ICL activity was determined in a phenylhydrazine-based assay as previously described [56,57], and product formation was followed at 324 nm using an extinction coefficient of 16.8 mM^-1^.cm^-1^. One unit of enzyme activity represents the formation of 1 µmol of glyoxylate-phenylhydrazone per minute. Specific activities were given as U/mg protein.

## Competing interests

The authors declare that they have no competing interests.

## Authors' contributions

GFP performed the bioinformatics analysis, laboratory experiments, and drafted the manuscript. NTAP participated in the laboratory experiments and in the writing of the manuscript draft. TRJ participated in the laboratory experiments. AR participated in the analysis of results and the manuscript preparation. RZNV participated in the bioinformatics analysis and reviewed the manuscript. NMM-R designed the project, supervised the research study, and prepared the manuscript. All the authors have read and approved the final manuscript.

## Supplementary Material

Additional file 1 Table S1 General features of the RNA-sequence mapped reads to *T. rubrum *reference genome. Click here for file

Additional file 2 Figure S1 Distribution of the expression levels of *T. rubrum *annotated genes measured using RPKM in each experimental condition. Genes with RPKM values greater than 1000 were grouped. Click here for file

Additional file 3 Figure S2 Volcano plot of the different experimental conditions. The log_2 _fold changes are plotted against the -log10 p-values for each of the analyzed genes. Click here for file

Additional file 4 Table S2 Genes modulated in response to acriflavine exposure Click here for file

Additional file 5 Table S3 Novel transcribed regions identified in the *T. rubrum *genome. Click here for file

Additional file 6 Table S4 Intergenic regions validated by the RT-PCR assay. Click here for file

Additional file 7 Table S5 Primers used in qPCR analysis. Click here for file
