# Supplementary material for: RNA-sequencing analysis of Trichophyton rubrum transcriptome in response to sublethal doses of acriflavine
Source: BMC Genomics. 2014 Oct 27;15(Suppl 7):S1. doi: 10.1186/1471-2164-15-S7-S1 (PMC4243288; doi:10.1186/1471-2164-15-S7-S1)
Supplement: Additional file 1 — Table S1 General features of the RNA-sequence mapped reads to T. rubrum reference genome. [file 1471-2164-15-S7-S1-S1.pdf]

Table S1

General features of the RNA-sequence mapped reads to the *T. rubrum* reference genome.

**Features**	**0 hour**	**3 hours**	**12 hours**	**24 hours**
Raw reads	50.901.088	67.868.533	43.067.783	40.179.474
High-quality reads	48.023.103	63.990.375	40.470.614	37.828.392
Mapped reads Bowtie	18.655.974	15.858.490	9.885.630	6.919.535
Mapped reads Tophat	15.428.064	12.899.690	7.669.542	5.647.140
Mapped reads 45pb	350.436	490.333	376.286	213.899
Mapped reads 40pb	239.726	476.602	339.681	230.067
Mapped reads 35pb	147.459	325.335	235.709	154.682
Mapped reads mitoDNA	1.095.756	2.571.798	1.728.431	989.246
Mapped reads repDNA*	9.237.127	20.247.164	11.585.272	13.816.158
**Total mapped reads (%)**	**61,90**	**62,43**	**59,68**	**59,01**

*repDNA – repeated DNA.
