# Supplementary figures and images for: RNA-sequencing analysis of Trichophyton rubrum transcriptome in response to sublethal doses of acriflavine
Source: BMC Genomics. 2014 Oct 27;15(Suppl 7):S1. doi: 10.1186/1471-2164-15-S7-S1 (PMC4243288; doi:10.1186/1471-2164-15-S7-S1)

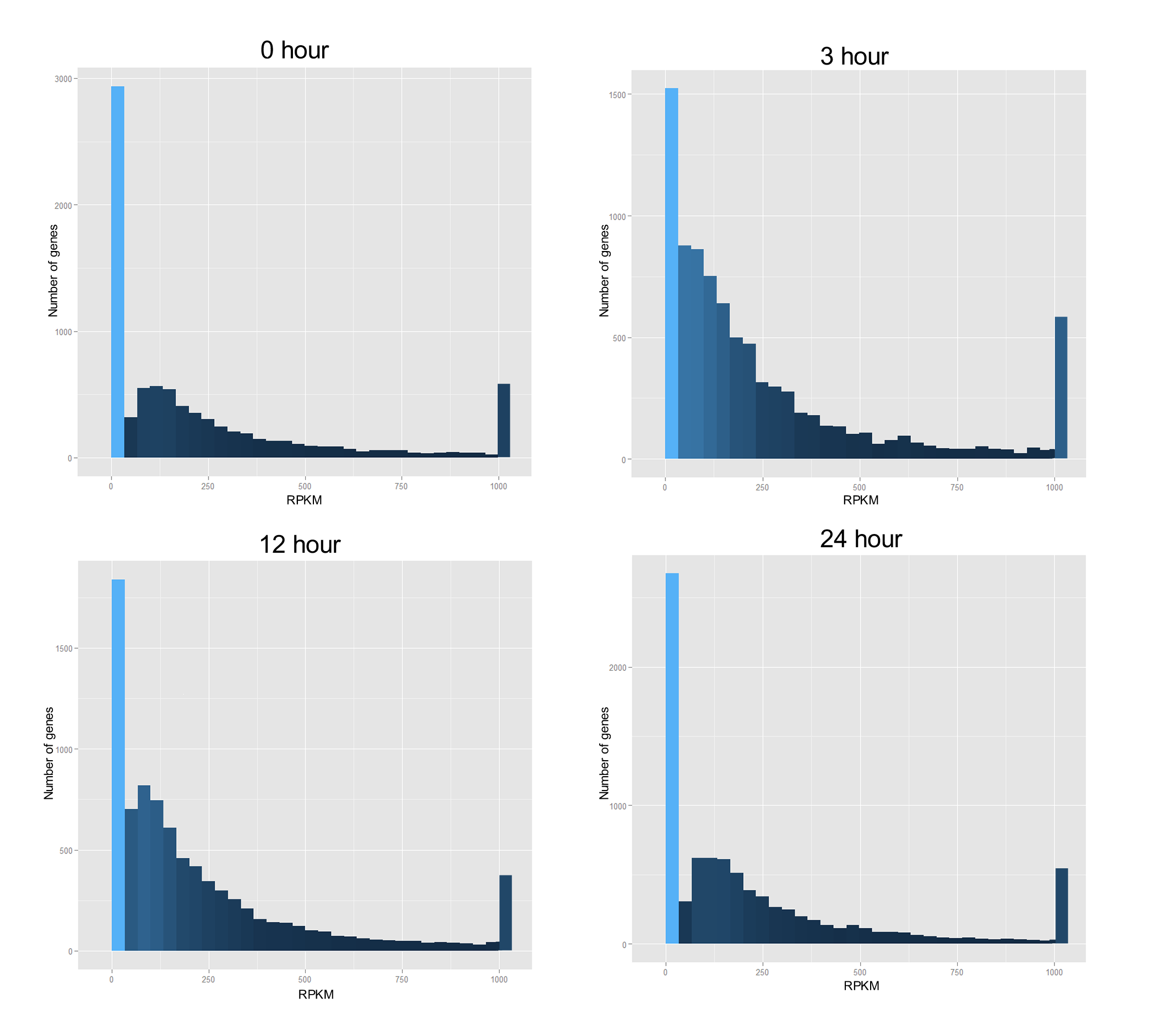

Supplement: Additional file 2 — Figure S1 Distribution of the expression levels of T. rubrum annotated genes measured using RPKM in each experimental condition. Genes with RPKM values greater than 1000 were grouped. [file 1471-2164-15-S7-S1-S2.tif]

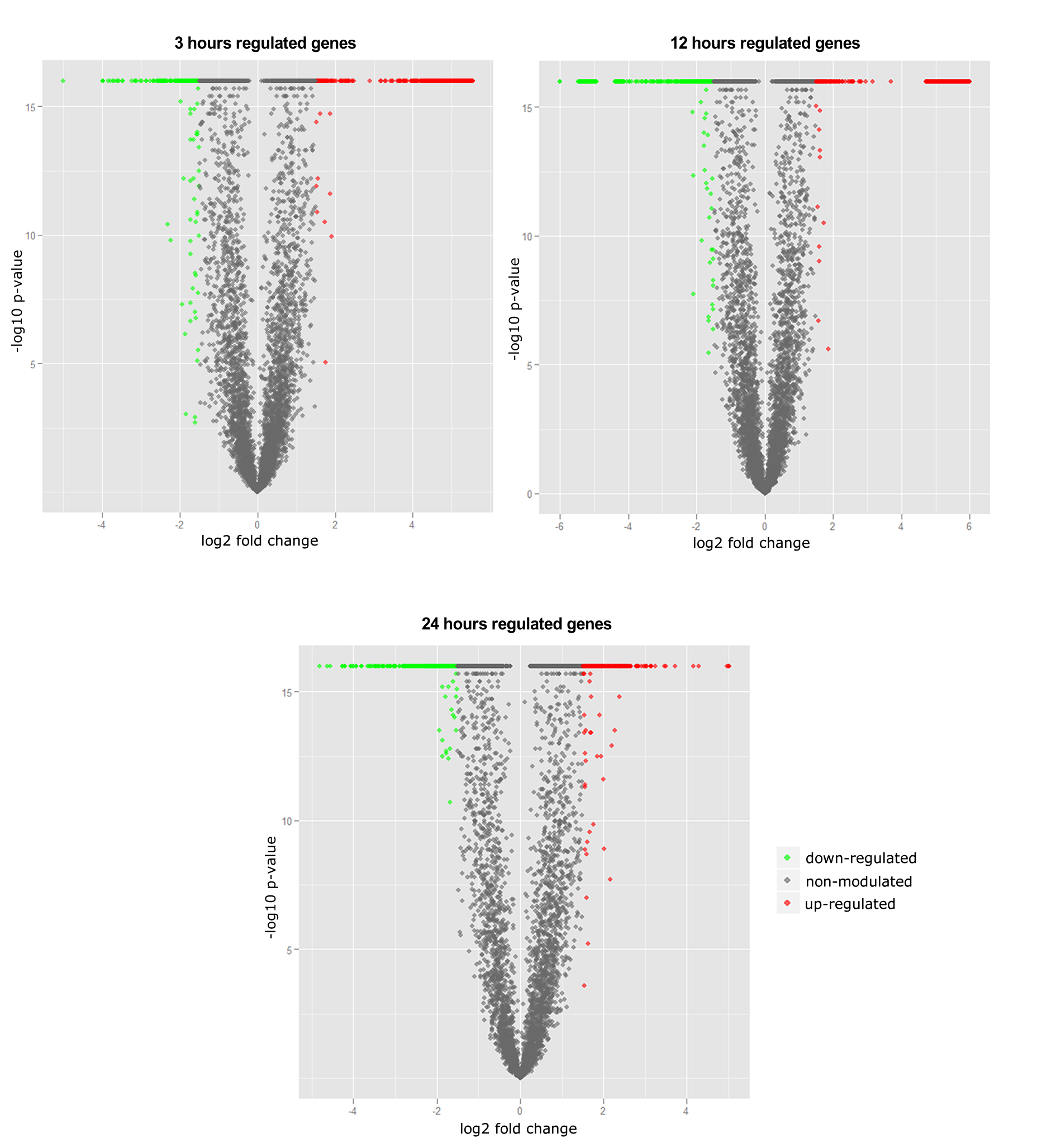

Supplement: Additional file 3 — Figure S2 Volcano plot of the different experimental conditions. The log2 fold changes are plotted against the -log10 p-values for each of the analyzed genes. [file 1471-2164-15-S7-S1-S3.tif]
