# Supplementary material for: RNA-sequencing analysis of Trichophyton rubrum transcriptome in response to sublethal doses of acriflavine
Source: BMC Genomics. 2014 Oct 27;15(Suppl 7):S1. doi: 10.1186/1471-2164-15-S7-S1 (PMC4243288; doi:10.1186/1471-2164-15-S7-S1)
Supplement: Additional file 4 — Table S2 Genes modulated in response to acriflavine exposure [file 1471-2164-15-S7-S1-S4.pdf]

Table S2

Genes modulated in response to acriflavine exposure.

3 hours

ID	3 hours	12 hours	24 hours	Gene Product Name
TERG_04160	9.51			hypothetical protein
TERG_03272	9.40			pre-mRNA branch site protein p14
TERG_07875	9.21			integral membrane protein
TERG_03023	9.15			hypothetical protein
TERG_02897	9.08			methylene-fatty-acyl-phospholipid synthase
TERG_01501	8.68			hypothetical protein
TERG_00732	8.59			hypothetical protein
TERG_03159	8.56			hypothetical protein
TERG_07957	8.45			hypothetical protein
TERG_03274	8.44			ammonium transporter MeaA
TERG_05514	8.25			oligosaccharyltransferase subunit ribophorin II
TERG_00854	8.22			hypothetical protein
TERG_00344	8.21			hypothetical protein
TERG_01219	8.15			malate/L-lactate dehydrogenase
TERG_08170	8.14			hypothetical protein
TERG_05137	8.11			microsomal dipeptidase
TERG_06006	7.87			hypothetical protein
TERG_08522	7.86			pyruvate dehydrogenase E1 B-subunit
TERG_08993	7.70			hypothetical protein
TERG_04487	7.69			hypothetical protein
TERG_06506	7.69			hypothetical protein
TERG_07879	7.64			hypothetical protein
TERG_00575	7.62			hypothetical protein
TERG_04531	7.53			hypothetical protein
TERG_02819	7.52			hypothetical protein
TERG_02385	7.51			hypothetical protein
TERG_02734	7.11			hypothetical protein
TERG_05478	6.96			chromatin remodeling complex subunit Arp5
TERG_07970	6.94			hypothetical protein
TERG_01291	6.62			hypothetical protein
TERG_00731	6.53			hypothetical protein
TERG_04504	2.44			hypothetical protein
TERG_07491	2.33			hypothetical protein
TERG_03388	2.18			hypothetical protein
TERG_00616	2.15			sodium transport ATPase
TERG_05363	2.14			hypothetical protein
TERG_04952	2.14			ABC transporter
TERG_04899	2.13			ubiquitin fusion protein
TERG_03037	2.03			dnaK-type molecular chaperone
TERG_01242	1.94			short chain dehydrogenase/reductase

TERG_06367	1.92	hypothetical protein
TERG_01659	1.92	hypothetical protein
TERG_07260	1.90	hypothetical protein
TERG_00807	1.88	hypothetical protein
TERG_08104	1.88	sodium transport ATPase
TERG_07921	1.86	adenosine 5'-phosphosulfate kinase
TERG_01973	1.86	mRNA cleavage and polyadenylation specificity factor complex subunit
TERG_08609	1.83	UV excision repair protein Rad2
TERG_06315	1.81	hypothetical protein
TERG_03283	1.80	hypothetical protein
TERG_03414	1.80	acetyl-CoA carboxylase
TERG_04521	1.77	hypothetical protein
TERG_04776	1.76	SH3 domain-containing protein
TERG_04662	1.75	protein disulfide isomerase
TERG_02028	1.75	FAS1 domain-containing protein
TERG_02891	1.72	hypothetical protein
TERG_00109	1.71	hypothetical protein
TERG_01871	1.71	acetyl-CoA acetyltransferase
TERG_03464	1.69	stress protein DDR48
TERG_08527	1.69	fatty acid synthase alpha subunit
TERG_00240	1.68	hypothetical protein
TERG_07456	1.68	hypothetical protein
TERG_05484	1.68	acyl-CoA dehydrogenase
TERG_07527	1.67	calnexin
TERG_05769	1.65	D-xylose reductase
TERG_01107	1.65	anthranilate synthase component I
TERG_08405	1.65	leucine aminopeptidase
TERG_01334	1.64	C6 finger domain-containing protein
TERG_07041	1.63	hypothetical protein
TERG_07997	1.63	acetamidase
TERG_07587	1.62	hypothetical protein
TERG_03618	1.61	endochitinase
TERG_07713	1.61	homoserine kinase
TERG_03062	1.61	DnaJ domain-containing protein
TERG_06505	1.61	hsp70-like protein
TERG_07946	1.61	3-ketodihydrosphingosine reductase
TERG_03158	1.60	hypothetical protein
TERG_03781	1.58	pyruvate dehydrogenase complex
TERG_01640	1.58	hypothetical protein
TERG_07764	1.58	protein transporter SEC61 alpha subunit
TERG_04096	1.57	SUMO activating enzyme
TERG_06007	1.57	coatomer subunit alpha
TERG_08065	1.57	membrane transporter
TERG_04400	1.56	monosaccharide transporter
TERG_05797	1.55	hypothetical protein

TERG_02221	1.54			proteasome subunit beta
TERG_03736	1.54			transcriptional corepressor Cyc8
TERG_07012	1.54			fibrillarin
TERG_05737	1.53			hypothetical protein
TERG_03224	1.52			choline oxidase
TERG_06511	1.52			mitochondrial escape protein 2
TERG_03487	1.51			translocation protein Sec62
TERG_02950	1.51			3',5'-bisphosphate nucleotidase
TERG_00633	1.50			40S ribosomal protein S9
TERG_07216	-1.50			hypothetical protein
TERG_02311	-1.51			hypothetical protein
TERG_03629	-1.51			hypothetical protein
TERG_07159	-1.52			protein kinase subdomain-containing protein
TERG_07987	-1.53			hypothetical protein
TERG_08557	-1.54			serine carboxypeptidase
TERG_02184	-1.56			hypothetical protein
TERG_06993	-1.58			hypothetical protein
TERG_06151	-1.60			pyridoxine kinase
TERG_06707	-1.62			arginase
TERG_06650	-1.65			hypothetical protein
TERG_08422	-1.71			hypothetical protein
TERG_02176	-1.79			small nuclear ribonucleoprotein LSM2
TERG_06333	-1.81			DNA ligase 4
TERG_08213	-1.86			hypothetical protein
TERG_01330	-1.90			hypothetical protein
TERG_08046	-2.01			hypothetical protein
TERG_05617	-2.04			hypothetical protein
TERG_06883	-2.22			hypothetical protein

Gene expression values are expressed in log_2 fold change between each time point and the control (0 hour).

12 hours

ID	3 hours	12 hours	24 hours	Gene Product Name
TERG_01685		9.98		glutathione transferase
TERG_05361		9.03		cript family protein
TERG_06053		8.37		catalase
TERG_08680		2.20		hypothetical protein
TERG_05284		2.18		hypothetical protein
TERG_06647		1.92		hypothetical protein
TERG_08979		1.91		CMGC/SRPK protein kinase
TERG_02369		1.81		MFS transporter
TERG_03896		1.78		mannose-6-phosphate isomerase
TERG_00020		1.72		SH3 domain-containing protein
TERG_08059		1.70		membrane transporter

TERG_04518	1.70	chromatin assembly factor 1 subunit C
TERG_01062	1.68	hypothetical protein
TERG_06500	1.62	mitochondrial carrier protein
TERG_08431	1.61	hypothetical protein
TERG_00614	1.61	dihydrodipicolinate synthetase
TERG_05175	1.60	hypothetical protein
TERG_07323	1.58	hypothetical protein
TERG_07672	1.56	GABA permease
TERG_01738	1.55	hypothetical protein
TERG_07876	1.55	hypothetical protein
TERG_08054	1.53	homoserine acetyltransferase
TERG_06504	1.53	hypothetical protein
TERG_07802	1.50	aerobactin siderophore biosynthesis protein iucB
TERG_08825	-1.61	hypothetical protein
TERG_02303	-1.62	ankyrin repeat protein
TERG_05558	-1.68	ornithine decarboxylase
TERG_07518	-1.72	histidyl-tRNA synthetase
TERG_08961	-1.81	hypothetical protein
TERG_05828	-1.83	DNA excision repair protein
TERG_06116	-1.84	indoleamine 2,3-dioxygenase
TERG_01550	-2.23	60S ribosomal protein L38
TERG_01496	-2.24	hypothetical protein
TERG_00298	-5.85	hypothetical protein

24 hours

ID	3 hours	12 hours	24 hours	Gene Product Name
TERG_00209			11.66	hypothetical protein
TERG_05855			9.73	ZIP zinc transporter
TERG_06788			9.66	zinc/iron transporter
TERG_02583			9.58	phosphate permease
TERG_05445			4.15	hypothetical protein
TERG_00911			3.46	hypothetical protein
TERG_00967			3.24	hypothetical protein
TERG_08353			2.97	cytochrome P450
TERG_01956			2.91	hypothetical protein
TERG_05270			2.85	C2H2 finger domain-containing protein
TERG_03172			2.79	sodium/phosphate symporter
TERG_07469			2.64	hypothetical protein
TERG_04107			2.58	hypothetical protein
TERG_02278			2.49	hypothetical protein
TERG_05615			2.43	hypothetical protein
TERG_00714			2.36	cell pattern formation-associated protein
TERG_08228			2.32	sphinganine hydroxylase
TERG_03762			2.21	malate dehydrogenase
TERG_08503			2.11	hypothetical protein

TERG_02809	1.99	hypothetical protein
TERG_07274	1.99	hypothetical protein
TERG_08982	1.96	hypothetical protein
TERG_00595	1.96	mitogen-activated protein kinase MAF1
TERG_07832	1.93	hypothetical protein
TERG_04855	1.90	hypothetical protein
TERG_01672	1.86	hypothetical protein
TERG_06759	1.85	C2H2 transcription factor
TERG_08380	1.84	hypothetical protein
TERG_01256	1.81	60S ribosomal protein L14
TERG_02818	1.77	hypothetical protein
TERG_04382	1.75	c-14 sterol reductase
TERG_03923	1.73	hypothetical protein
TERG_08090	1.73	plasma membrane iron permease
TERG_01623	1.72	MFS transporter
TERG_05178	1.69	nuclear and cytoplasmic polyadenylated RNA-binding protein pub1
TERG_02198	1.68	CAMK protein kinase
TERG_07681	1.68	hypothetical protein
TERG_08023	1.68	NAD(P) transhydrogenase
TERG_01994	1.67	hypothetical protein
TERG_07555	1.67	CAMKK protein kinase
TERG_04042	1.66	serine/threonine protein kinase
TERG_00838	1.65	zinc finger transcription factor pacC
TERG_07236	1.65	hypothetical protein
TERG_05381	1.64	Na(+)/H(+) antiporter
TERG_07199	1.64	hypothetical protein
TERG_05971	1.64	ribonucleotide reductase small subunit RnrA
TERG_08141	1.63	hypothetical protein
TERG_05524	1.61	phospho-2-dehydro-3-deoxyheptonate aldolase
TERG_00243	1.60	hypothetical protein
TERG_00976	1.60	serine hydroxymethyltransferase
TERG_00625	1.59	Glucanosyltransferase
TERG_00393	1.56	hypothetical protein
TERG_05518	1.54	short chain dehydrogenase
TERG_01127	1.54	glucan synthase
TERG_05832	1.54	serine/threonine protein phosphatase
TERG_01731	1.53	hypothetical protein
TERG_00802	1.52	hypothetical protein
TERG_07810	1.50	hypothetical protein
TERG_02450	-1.50	phosphoserine phosphatase
TERG_04726	-1.50	nuclear transcription factor Y subunit B-7
TERG_03684	-1.51	stress activated MAP kinase interacting protein
TERG_00174	-1.51	anion-transporting ATPase
TERG_03307	-1.52	hypothetical protein
TERG_02063	-1.52	hypothetical protein

TERG_00278	-1.53	electron transfer flavoprotein-ubiquinone oxidoreductase
TERG_03263	-1.53	GTPase activating protein Gyp1
TERG_04363	-1.53	cysteine synthase
TERG_05265	-1.53	DEAD/DEAH box helicase
TERG_03221	-1.53	C6 transcription factor
TERG_01165	-1.54	40S ribosomal protein S10-A
TERG_03102	-1.54	sterol 24-C-methyltransferase
TERG_02603	-1.54	hypothetical protein
TERG_02788	-1.56	DNA-directed RNA polymerase I
TERG_06113	-1.56	hypothetical protein
TERG_02532	-1.56	hypothetical protein
TERG_01588	-1.56	60S ribosome biogenesis protein Mak11
TERG_02237	-1.56	30S ribosomal protein S28e
TERG_03804	-1.58	dihydroxyacid dehydratase
TERG_04983	-1.58	histone acetyltransferase E
TERG_08336	-1.59	MFS multidrug transporter
TERG_02886	-1.59	hypothetical protein
TERG_01163	-1.59	hypothetical protein
TERG_04512	-1.60	transcriptional regulator Cwf13/SkiP
TERG_07525	-1.60	methionine aminopeptidase
TERG_07058	-1.61	hypothetical protein
TERG_04852	-1.61	U-box domain-containing protein
TERG_02283	-1.61	MFS transporter
TERG_02644	-1.62	hypothetical protein
TERG_02325	-1.62	T-complex protein 1
TERG_01603	-1.63	hypothetical protein
TERG_05257	-1.63	integral membrane protein
TERG_03494	-1.64	hypothetical protein
TERG_03654	-1.65	NADH-cytochrome b5 reductase
TERG_06187	-1.65	nucleolar ATPase Kre33
TERG_00903	-1.65	SNF7 family protein
TERG_04139	-1.65	hypothetical protein
TERG_04692	-1.66	replication factor A 1
TERG_04951	-1.66	hypothetical protein
TERG_06398	-1.66	Hsp90 co-chaperone Cdc37
TERG_06090	-1.67	C2H2 transcription factor
TERG_00008	-1.68	MFS phospholipid transporter
TERG_00075	-1.70	hypothetical protein
TERG_03516	-1.73	mitochondrial dicarboxylate Carrier
TERG_03933	-1.73	ABC metal ion transporter
TERG_07779	-1.75	LCCL domain-containing protein
TERG_00961	-1.75	T-complex protein 1
TERG_01310	-1.75	nascent polypeptide-associated complex subunit beta
TERG_06103	-1.76	uroporphyrinogen decarboxylase
TERG_06846	-1.77	IMP dehydrogenase

TERG_05827	-1.77	sphingolipid long chain base-responsive protein PIL1
TERG_08367	-1.79	aconitate hydratase
TERG_07897	-1.79	CCAAT-box-binding transcription factor
TERG_06207	-1.80	hypothetical protein
TERG_02491	-1.81	glutaryl-CoA dehydrogenase
TERG_06177	-1.81	peroxisomal carrier protein
TERG_04807	-1.81	Aminotransferase
TERG_01281	-1.83	malate synthase
TERG_01597	-1.84	small nucleolar ribonucleoprotein complex subunit
TERG_06451	-1.84	nucleolus protein required for cell viability
TERG_02031	-1.84	methylenetetrahydrofolate dehydrogenase
TERG_07639	-1.85	atypical/ABC1/ABC1-B protein kinase
TERG_03202	-1.86	eukaryotic translation initiation factor 3 subunit C
TERG_00217	-1.88	Ubiquitin
TERG_03935	-1.89	hypothetical protein
TERG_01002	-1.90	chaperone dnaK
TERG_02979	-1.91	Delta(24(24(1)))-sterol reductase
TERG_07157	-1.91	hypothetical protein
TERG_03262	-1.92	dimethyladenosine transferase dimethyltransferase
TERG_00069	-1.93	Fumarylacetoacetase
TERG_02078	-1.93	thiamine biosynthesis protein Nmt1
TERG_02172	-1.95	cytochrome P450 monoxygenase
TERG_00232	-1.95	rRNA processing protein Nop9
TERG_02833	-1.95	hypothetical protein
TERG_06078	-1.96	eukaryotic translation initiation factor 3 subunit
TERG_06147	-2.02	rubredoxin-NAD(+) reductase
TERG_00604	-2.02	nucleolar protein 16
TERG_06792	-2.03	hypothetical protein
TERG_04686	-2.04	porphobilinogen synthase
TERG_07027	-2.08	MFS drug transporter
TERG_03292	-2.10	NADH-ubiquinone oxidoreductase 24 kDa subunit
TERG_03360	-2.10	prolyl peptidase
TERG_00181	-2.10	nucleolar protein NOP58
TERG_06522	-2.10	mitochondrial cytochrome b2
TERG_00281	-2.16	AGC/RSK protein kinase
TERG_07140	-2.17	serine/threonine protein kinase
TERG_07585	-2.18	Emp24/gp25L/p24 membrane trafficking protein
TERG_06452	-2.20	purine nucleoside phosphorylase
TERG_06573	-2.25	calcineurin regulatory subunit B
TERG_05058	-2.26	thiamine pyrophosphokinase
TERG_05388	-2.27	ribosome biogenesis protein
TERG_06783	-2.28	nucleolar GTP-binding protein
TERG_00820	-2.30	MFS multidrug resistance transporter
TERG_03780	-2.53	hypothetical protein
TERG_03455	-2.70	isochorismatase family hydrolase
TERG_00067	-2.89	hypothetical protein

TERG_00546			-3.04	Glutaredoxin
TERG_04238			-3.38	cation-transporting ATPase
TERG_03385			-9.43	hypothetical protein

3 and 12 hours

ID	3 hours	12 hours	24 hours	Gene Product Name
TERG_00500	8.43	9.57		hypothetical protein
TERG_02069	8.75	9.54		hypothetical protein
TERG_07426	8.55	9.25		hypothetical protein
TERG_05520	8.63	8.79		hypothetical protein
TERG_03127	7.32	8.70		hypothetical protein
TERG_08976	8.46	8.43		hypothetical protein
TERG_03764	8.02	8.35		hypothetical protein
TERG_08259	7.02	8.16		hypothetical protein
TERG_01658	3.18	3.16		hypothetical protein
TERG_00856	2.35	2.56		hypothetical protein
TERG_00580	3.31	2.55		7-dehydrocholesterol reductase
TERG_02001	1.86	2.49		oligopeptidase
TERG_04765	2.30	2.35		hypothetical protein
TERG_01507	2.37	2.29		hypothetical protein
TERG_08264	1.64	2.20		mannitol dehydrogenase
TERG_06757	2.89	2.18		hypothetical protein
TERG_07928	2.18	2.11		hypothetical protein
TERG_00624	2.44	2.07		actin polymerization protein Bzz1
TERG_01122	1.94	1.95		hypothetical protein
TERG_03597	1.78	1.93		integral membrane protein
TERG_07566	2.48	1.88		hypothetical protein
TERG_00250	1.62	1.78		N-acetylglucosamine-phosphate mutase
TERG_04125	1.71	1.73		citrate synthase
TERG_02140	1.70	1.64		bZIP transcription factor
TERG_08388	1.71	1.56		hypothetical protein
TERG_04103	-1.87	-1.51		hypothetical protein
TERG_06250	-1.81	-1.54		hypothetical protein
TERG_03303	-1.52	-1.73		extracellular 3-ketosteroid 1-dehydrogenase
TERG_02012	-1.67	-2.03		hypothetical protein
TERG_08231	-2.21	-2.21		hypothetical protein
TERG_01981	-1.78	-2.24		hypothetical protein

3 and 24 hours

ID	3 hours	12 hours	24 hours	Gene Product Name

TERG_05040	1.74	3.01	hypothetical protein
TERG_03234	1.80	2.51	hypothetical protein
TERG_02447	2.25	2.30	ATP sulphurylase
TERG_07631	1.74	2.10	hypothetical protein
TERG_02787	2.34	2.00	protein kinase subdomain-containing protein
TERG_07595	1.61	1.62	hypothetical protein
TERG_08288	1.88	1.59	ATP-citrate synthase subunit 1
TERG_00762	1.55	1.58	vesicle-fusing ATPase
TERG_01443	1.80	1.52	ABC multidrug transporter
TERG_00121	-1.72	-1.53	FAD dependent oxidoreductase
TERG_06553	-1.81	-1.55	hypothetical protein
TERG_06270	-1.77	-1.55	hypothetical protein
TERG_00865	1.78	-1.63	hypothetical protein
TERG_04290	-1.78	-1.68	D-arabinitol dehydrogenase
TERG_03368	-1.69	-1.72	hypothetical protein
TERG_08619	-2.76	-1.72	siderophore iron transporter
TERG_03605	-1.54	-1.83	mitochondrial ribosomal protein L16
TERG_03708	-1.90	-1.88	hypothetical protein
TERG_03364	-2.00	-1.99	hypothetical protein
TERG_05899	-1.69	-2.20	hypothetical protein
TERG_03363	-1.66	-2.46	GABA permease
TERG_08620	-2.25	-2.61	siderophore iron transporter mirB
TERG_07539	-1.64	-3.49	hypothetical protein
TERG_08906	-1.62	-8.74	hypothetical protein

12 and 24 hours

ID	3 hours	12 hours	24 hours	Gene Product Name
TERG_00698		9.42	10.63	L-ornithine 5-monooxygenase
TERG_02132		8.23	8.71	hypothetical protein
TERG_02242		2.82	5.02	hypothetical protein
TERG_03174		3.69	4.97	siderochrome-iron transporter Sit1
TERG_00511		2.54	3.72	hypothetical protein
TERG_00697		2.79	3.51	non-ribosomal peptide synthetase
TERG_07801		2.60	3.13	ABC multidrug transporter Mdr4
TERG_04228		1.77	3.13	hypothetical protein
TERG_06267		1.61	3.13	hypothetical protein
TERG_03145		1.61	3.03	hypothetical protein
TERG_00509		1.89	2.78	hypothetical protein
TERG_08201		1.55	2.63	serine protease
TERG_04240		2.12	2.59	hypothetical protein
TERG_08102		1.77	2.45	3-isopropylmalate dehydrogenase A
TERG_02177		1.55	2.43	hypothetical protein
TERG_04862		2.26	2.37	C6 sexual development transcription factor NosA
TERG_02454		2.02	2.30	deoxyribose-phosphate aldolase
TERG_02984		1.83	2.28	cytochrome P450 51

TERG_04234	1.79	2.24	hypothetical protein
TERG_00520	2.03	2.23	hypothetical protein
TERG_01494	2.10	2.14	hypothetical protein
TERG_07241	1.75	2.06	saccharopine dehydrogenase
TERG_08983	1.55	2.00	hypothetical protein
TERG_05651	1.79	1.95	hypothetical protein
TERG_02367	1.54	1.88	hypothetical protein
TERG_00830	1.60	1.57	hypothetical protein
TERG_04105	1.91	1.57	cyclin domain-containing protein
TERG_00456	1.64	1.52	cysteine dioxygenase
TERG_04323	-1.60	-1.56	hypothetical protein
TERG_04194	-1.61	-1.72	acyltransferase
TERG_02256	-1.67	-1.77	hypothetical protein
TERG_08130	-1.70	-1.77	ABC transporter
TERG_07346	-1.62	-1.79	tRNA ligase
TERG_01811	-1.92	-1.85	lipase
TERG_03625	-1.62	-1.94	hypothetical protein
TERG_02174	-2.32	-1.96	hypothetical protein
TERG_08278	-1.84	-1.99	serine/threonine protein kinase
TERG_00959	-1.94	-2.04	hypothetical protein
TERG_04020	-2.18	-2.06	polymerase
TERG_08613	-1.70	-2.14	multidrug resistance protein
TERG_01644	-1.70	-2.15	SNF2 family helicase/ATPase
TERG_02942	-1.78	-2.22	hypothetical protein
TERG_05450	-2.16	-2.23	acetyltransferase
TERG_07215	-1.90	-2.26	hypothetical protein
TERG_02029	-1.63	-2.31	hypothetical protein
TERG_07906	-1.72	-2.37	hypothetical protein
TERG_05153	-1.89	-2.37	MFS transporter
TERG_00068	-2.24	-2.45	homogentisate 1,2-dioxygenase
TERG_03380	-1.99	-2.67	FKBP-type peptidyl-prolyl cis-trans isomerase
TERG_00825	-2.18	-2.69	isocitrate lyase
TERG_04612	-1.63	-2.76	branched-chain-amino-acid aminotransferase
TERG_05668	-1.69	-2.80	hypothetical protein
TERG_05816	-3.15	-2.82	hypothetical protein
TERG_06938	-1.95	-3.01	zinc metallopeptidase
TERG_03529	-1.78	-3.11	hypothetical protein
TERG_05332	-1.61	-3.12	mitochondrial carrier protein
TERG_03337	-2.58	-3.16	hypothetical protein
TERG_07214	-1.78	-3.29	DlpA domain-containing protein
TERG_00066	-2.58	-3.32	4-hydroxyphenylpyruvate dioxygenase
TERG_01346	-2.83	-3.40	lipase/serine esterase
TERG_01609	-2.80	-3.66	Na/K ATPase alpha 1 subunit

3, 12 and 24 hours

ID	3 hours	12 hours	24 hours	Gene Product Name
TERG_01497	5.50	5.38	6.39	protein kinase subdomain-containing protein
TERG_05744	2.26	2.76	4.29	GTP-binding protein EsdC
TERG_07143	4.09	2.94	3.15	potassium/sodium efflux P-type ATPase
TERG_00710	2.22	1.71	3.03	hypothetical protein
TERG_03678	1.56	1.61	2.83	hypothetical protein
TERG_01252	1.70	2.45	2.80	catalase
TERG_08286	1.90	1.96	2.60	hypothetical protein
TERG_00501	1.75	2.00	2.54	hypothetical protein
TERG_06727	1.80	2.25	2.37	homoserine dehydrogenase
TERG_08287	1.98	1.75	2.34	ATP-citrate-lyase
TERG_01444	2.31	1.71	1.81	nonribosomal peptide synthase
TERG_02610	-1.53	-2.86	-1.54	YjgH family protein
TERG_02199	-2.36	-2.62	-1.70	glutamate carboxypeptidase
TERG_02261	-1.54	-1.53	-1.90	pyruvate formate lyase activating enzyme
TERG_05655	-1.69	-1.79	-1.93	AN1 zinc finger protein
TERG_05039	-1.72	-2.37	-1.96	hypothetical protein
TERG_06220	-2.25	-1.86	-1.97	hypothetical protein
TERG_04458	-1.62	-1.95	-1.99	inosine triphosphate pyrophosphatase
TERG_04915	-1.60	-2.09	-2.06	alpha/beta hydrolase
TERG_07351	-1.85	-2.06	-2.07	hypothetical protein
TERG_03166	-1.67	-1.68	-2.14	hypothetical protein
TERG_05625	-2.09	-1.97	-2.15	hypothetical protein
TERG_06909	-1.95	-1.93	-2.19	succinate-semialdehyde dehydrogenase
TERG_02196	-1.72	-1.75	-2.25	l-allo-threonine aldolase
TERG_00402	-1.60	-2.10	-2.25	multidrug resistance protein
TERG_00955	-1.66	-1.79	-2.29	ABC efflux transporter
TERG_05917	-1.56	-1.90	-2.30	hypothetical protein
TERG_07008	-1.90	-1.77	-2.33	acetylornithine deacetylase
TERG_06730	-1.68	-2.15	-2.36	hypothetical protein
TERG_01893	-1.92	-2.36	-2.39	hypothetical protein
TERG_02528	-2.06	-2.82	-2.42	arsenic methyltransferase Cyt19
TERG_00709	-2.38	-2.22	-2.47	hypothetical protein
TERG_05106	-3.12	-2.61	-2.56	hypothetical protein
TERG_08622	-2.52	-1.56	-2.56	transferase
TERG_00073	-1.88	-1.85	-2.61	NADH-dependent flavin oxidoreductase
TERG_06161	-2.42	-2.26	-2.66	multidrug resistance protein
TERG_06445	-2.30	-3.77	-2.74	hypothetical protein
TERG_05849	-1.84	-1.59	-2.80	thioredoxin reductase
TERG_07427	-2.58	-2.45	-2.82	hypothetical protein
TERG_03855	-1.92	-2.48	-2.90	hypothetical protein
TERG_05409	-1.84	-2.94	-2.98	FAD dependent oxidoreductase
TERG_00523	-2.40	-3.90	-3.01	hypothetical protein
TERG_02538	-1.99	-2.84	-3.18	hypothetical protein
TERG_06701	-2.68	-3.38	-3.19	gamma-glutamyltranspeptidase
TERG_00227	-1.72	-3.05	-3.25	glutathione S-transferase

TERG_03443	-2.66	-2.40	-3.30	hypothetical protein
TERG_04303	-3.98	-3.33	-3.31	hypothetical protein
TERG_07408	-2.51	-3.14	-3.38	hypothetical protein
TERG_03712	-2.00	-2.70	-3.44	hypothetical protein
TERG_04146	-2.40	-2.77	-3.47	FAD binding domain-containing protein
TERG_03352	-2.23	-2.74	-3.48	tyrosine decarboxylase
TERG_00736	-3.14	-3.47	-3.54	hypothetical protein
TERG_05677	-2.24	-2.64	-3.59	hypothetical protein
TERG_02400	-1.93	-3.01	-3.62	amino acid permease
TERG_06679	-2.67	-3.26	-3.82	hypothetical protein
TERG_07734	-2.47	-4.04	-3.93	O-methyltransferase
TERG_01338	-3.01	-3.77	-3.93	hypothetical protein
TERG_01401	-3.78	-2.15	-4.06	high affinity copper transporter
TERG_02214	-2.59	-3.51	-4.20	zinc carboxypeptidase
TERG_03009	-1.83	-3.11	-4.21	hypothetical protein
TERG_07139	-2.54	-4.14	-4.26	hypothetical protein
TERG_06466	-2.97	-3.98	-4.56	hypothetical protein
TERG_08129	-3.48	-3.71	-4.64	amino acid permease
TERG_07861	-2.54	-3.61	-4.81	subtilase
TERG_01859	-3.58	-2.32	-9.11	hypothetical protein
TERG_01454	-3.56	-2.81	-9.47	microsomal dipeptidase
TERG_05545	-2.86	-4.39	-10.50	hypothetical protein
TERG_05854	-3.63	-3.98	-11.20	beta-lactamase
TERG_02517	-3.70	-5.45	-12.32	hypothetical protein
