# Supplementary material for: RNA-sequencing analysis of Trichophyton rubrum transcriptome in response to sublethal doses of acriflavine
Source: BMC Genomics. 2014 Oct 27;15(Suppl 7):S1. doi: 10.1186/1471-2164-15-S7-S1 (PMC4243288; doi:10.1186/1471-2164-15-S7-S1)
Supplement: Additional file 5 — Table S3 Novel transcribed regions identified in the T. rubrum genome. [file 1471-2164-15-S7-S1-S5.pdf]

Table S3

Novel transcribed regions identified in the *T. rubrum* genome.

Chromosome	Start	End	Name
Supercontig 1	48064	48616	TCONS_0000001
Supercontig 1	298934	300985	TCONS_0000002
Supercontig 1	301280	303009	TCONS_0000003
Supercontig 1	799814	800845	TCONS_0000004
Supercontig 1	938415	938547	TCONS_0000005
Supercontig 1	1367238	1368522	TCONS_0000006
Supercontig 1	1408696	1409013	TCONS_0000007
Supercontig 1	1409094	1409599	TCONS_0000008
Supercontig 1	1476100	1476481	TCONS_0000009
Supercontig 1	1555889	1557125	TCONS_0000010
Supercontig 1	1791520	1796216	TCONS_0000011
Supercontig 1	1796270	1798287	TCONS_0000012
Supercontig 1	1798387	1799582	TCONS_0000013
Supercontig 1	1873104	1873717	TCONS_0000014
Supercontig 1	2041983	2043441	TCONS_0000015
Supercontig 1	2044122	2045389	TCONS_0000016
Supercontig 1	2299147	2300245	TCONS_0000017
Supercontig 1	2300496	2302117	TCONS_0000018
Supercontig 1	2508283	2509291	TCONS_0000019
Supercontig 1	2678421	2679571	TCONS_0000020
Supercontig 1	3209772	3211965	TCONS_0000021
Supercontig 1	3266687	3266873	TCONS_0000022
Supercontig 1	3267258	3267948	TCONS_0000023
Supercontig 1	3336795	3337566	TCONS_0000024
Supercontig 1	3359397	3359573	TCONS_0000025
Supercontig 1	3445287	3446677	TCONS_0000026
Supercontig 1	3457208	3458064	TCONS_0000027
Supercontig 1	3479455	3480536	TCONS_0000028
Supercontig 1	3592386	3593403	TCONS_0000029
Supercontig 1	3662538	3663660	TCONS_0000030
Supercontig 1	3664191	3665120	TCONS_0000031
Supercontig 1	3693100	3693925	TCONS_0000032
Supercontig 1	3725014	3726205	TCONS_0000033

Supercontig 1	3809663	3811173	TCONS_0000034
Supercontig 1	3813300	3813899	TCONS_0000035
Supercontig 2	147611	148790	TCONS_0000036
Supercontig 2	203278	203543	TCONS_0000037
Supercontig 2	340504	341386	TCONS_0000038
Supercontig 2	417920	420407	TCONS_0000039
Supercontig 2	607922	609286	TCONS_0000040
Supercontig 2	856924	857069	TCONS_0000041
Supercontig 2	886204	886867	TCONS_0000042
Supercontig 2	1377827	1378243	TCONS_0000043
Supercontig 2	1505373	1506549	TCONS_0000044
Supercontig 2	1506616	1507627	TCONS_0000045
Supercontig 2	1768942	1769399	TCONS_0000046
Supercontig 2	1837620	1839226	TCONS_0000047
Supercontig 2	1957884	1959002	TCONS_0000048
Supercontig 2	1961914	1963451	TCONS_0000049
Supercontig 2	2099017	2101500	TCONS_0000050
Supercontig 2	2454861	2456392	TCONS_0000051
Supercontig 2	2471359	2472333	TCONS_0000052
Supercontig 2	2554188	2554829	TCONS_0000053
Supercontig 2	2641970	2643150	TCONS_0000054
Supercontig 2	2661721	2662739	TCONS_0000055
Supercontig 2	2839322	2840137	TCONS_0000056
Supercontig 2	2934368	2936039	TCONS_0000057
Supercontig 3	140549	142143	TCONS_0000058
Supercontig 3	323632	324731	TCONS_0000059
Supercontig 3	651527	652970	TCONS_0000060
Supercontig 3	699153	700151	TCONS_0000061
Supercontig 3	764718	765180	TCONS_0000062
Supercontig 3	857288	857858	TCONS_0000063
Supercontig 3	960823	962430	TCONS_0000064
Supercontig 3	964150	965407	TCONS_0000065
Supercontig 3	1713983	1714304	TCONS_0000066
Supercontig 3	1776378	1778361	TCONS_0000067
Supercontig 3	1863042	1863393	TCONS_0000068
Supercontig 3	1871126	1871278	TCONS_0000069
Supercontig 3	1897548	1897927	TCONS_0000070

Supercontig 3	1931792	1932025	TCONS_0000071
Supercontig 3	2045468	2046392	TCONS_0000072
Supercontig 3	2067117	2067805	TCONS_0000073
Supercontig 3	2107543	2108022	TCONS_0000074
Supercontig 3	2175938	2177441	TCONS_0000075
Supercontig 3	2177889	2178964	TCONS_0000076
Supercontig 3	2636914	2637823	TCONS_0000077
Supercontig 3	2806998	2807186	TCONS_0000078
Supercontig 3	2844388	2845576	TCONS_0000079
Supercontig 4	26353	27904	TCONS_0000080
Supercontig 4	189385	190925	TCONS_0000081
Supercontig 4	199395	200312	TCONS_0000082
Supercontig 4	438436	439285	TCONS_0000083
Supercontig 4	465073	465761	TCONS_0000084
Supercontig 4	488083	488763	TCONS_0000085
Supercontig 4	489176	490233	TCONS_0000086
Supercontig 4	707665	708598	TCONS_0000087
Supercontig 4	708662	709746	TCONS_0000088
Supercontig 4	709814	710802	TCONS_0000089
Supercontig 4	858143	862210	TCONS_0000090
Supercontig 4	981001	981557	TCONS_0000091
Supercontig 4	1015253	1017131	TCONS_0000092
Supercontig 4	1222875	1224851	TCONS_0000093
Supercontig 4	1266657	1268212	TCONS_0000094
Supercontig 4	1316635	1318758	TCONS_0000095
Supercontig 4	1544942	1545282	TCONS_0000096
Supercontig 4	1746467	1748432	TCONS_0000097
Supercontig 4	2076789	2077546	TCONS_0000098
Supercontig 5	27338	28685	TCONS_0000099
Supercontig 5	393653	394735	TCONS_0000100
Supercontig 5	404139	404405	TCONS_0000101
Supercontig 5	405102	405258	TCONS_0000102
Supercontig 5	408714	408904	TCONS_0000103
Supercontig 5	514436	515444	TCONS_0000104
Supercontig 5	669448	670234	TCONS_0000105
Supercontig 5	856950	858532	TCONS_0000106
Supercontig 5	1327943	1328639	TCONS_0000107

Supercontig 5	1420535	1420665	TCONS_0000108
Supercontig 5	1461800	1463517	TCONS_0000109
Supercontig 6	38867	39039	TCONS_0000110
Supercontig 6	492533	494917	TCONS_0000111
Supercontig 6	628222	628707	TCONS_0000112
Supercontig 6	895185	895624	TCONS_0000113
Supercontig 6	895749	896422	TCONS_0000114
Supercontig 6	913532	914910	TCONS_0000115
Supercontig 6	942333	943753	TCONS_0000116
Supercontig 6	1157984	1158724	TCONS_0000117
Supercontig 6	1171751	1173177	TCONS_0000118
Supercontig 6	1381925	1383088	TCONS_0000119
Supercontig 7	114817	115152	TCONS_0000120
Supercontig 7	198295	199882	TCONS_0000121
Supercontig 7	383064	384377	TCONS_0000122
Supercontig 7	723404	723608	TCONS_0000123
Supercontig 7	777300	778230	TCONS_0000124
Supercontig 7	790382	791312	TCONS_0000125
Supercontig 7	956076	956597	TCONS_0000126
Supercontig 7	1062948	1064051	TCONS_0000127
Supercontig 7	1222517	1224995	TCONS_0000128
Supercontig 7	1344492	1346228	TCONS_0000129
Supercontig 8	120034	120315	TCONS_0000130
Supercontig 8	364982	366734	TCONS_0000131
Supercontig 8	566522	566846	TCONS_0000132
Supercontig 8	649014	649760	TCONS_0000133
Supercontig 9	31148	33353	TCONS_0000134
Supercontig 9	177861	179018	TCONS_0000135
Supercontig 9	188938	189877	TCONS_0000136
Supercontig 9	408628	409810	TCONS_0000137
Supercontig 9	693054	693457	TCONS_0000138
Supercontig 9	695119	696616	TCONS_0000139
Supercontig 9	796435	797172	TCONS_0000140
Supercontig 10	14991	15726	TCONS_0000141
Supercontig 10	313286	315010	TCONS_0000142
Supercontig 10	422178	422364	TCONS_0000143
Supercontig 10	422427	422596	TCONS_0000144

Supercontig 10	422891	423123	TCONS_0000145
Supercontig 10	512927	513485	TCONS_0000146
Supercontig 10	536172	538315	TCONS_0000147
Supercontig 10	576724	577559	TCONS_0000148
Supercontig 10	621479	623460	TCONS_0000149
Supercontig 10	643535	645536	TCONS_0000150
Supercontig 10	724086	725892	TCONS_0000151
Supercontig 11	168881	170283	TCONS_0000152
Supercontig 11	178039	179792	TCONS_0000153
Supercontig 11	258373	260061	TCONS_0000154
Supercontig 12	8446	9710	TCONS_0000155
Supercontig 12	452574	453133	TCONS_0000156
Supercontig 18	13786	14642	TCONS_0000157
Supercontig 18	14724	15109	TCONS_0000158
Supercontig 18	15182	15938	TCONS_0000159
Supercontig 18	16130	16552	TCONS_0000160
Supercontig 18	16624	17180	TCONS_0000161
