# Supplementary material for: RNA-sequencing analysis of Trichophyton rubrum transcriptome in response to sublethal doses of acriflavine
Source: BMC Genomics. 2014 Oct 27;15(Suppl 7):S1. doi: 10.1186/1471-2164-15-S7-S1 (PMC4243288; doi:10.1186/1471-2164-15-S7-S1)
Supplement: Additional file 6 — Table S4 Intergenic regions validated by the RT-PCR assay. [file 1471-2164-15-S7-S1-S6.pdf]

Table S4

Intergenic regions validated by the RT-PCR assay.

Chromosome	Start	End	Name	Primer (5' - 3')	Amplicon (bp)
Supercontig 1	3693100	3693925	TCONS_0000032	FW: GTAATGGCCGAAGGGAAACT<br>REV: GGCCTCTGCAGAATTGCTT	211
Supercontig 2	1957884	1959002	TCONS_0000048	FW: CCAATCAACACTCCAAAACG<br>REV: GCCAATTGAACAGTGGAGGT	616
Supercontig 4	438436	439285	TCONS_0000083	FW: CCCACCTGCGAATATACGAT<br>REV: CTTGTCATCCCATGCAAATG	206
Supercontig 5	1327943	1328639	TCONS_0000107	FW: GAGGAAACCGCAAAAGTGAC<br>REV: ACAATTGCCAGGGTAACACC	197
Supercontig 9	695119	696616	TCONS_0000139	FW: AGAAGGATGAATGGCACTGG<br>REV: CAGGTTCAGTTTGGCATGAA	342
Supercontig 8	332979	334049	TERG_06637<br>(actin)	FWD: GAATCTCCCCCCTCATGCTT<br>REV: GCCTGGGCTTCCGACACT	198
