# Supplementary material for: RNA-sequencing analysis of Trichophyton rubrum transcriptome in response to sublethal doses of acriflavine
Source: BMC Genomics. 2014 Oct 27;15(Suppl 7):S1. doi: 10.1186/1471-2164-15-S7-S1 (PMC4243288; doi:10.1186/1471-2164-15-S7-S1)
Supplement: Additional file 7 — Table S5 Primers used in qPCR analysis. [file 1471-2164-15-S7-S1-S7.pdf]

Table S5

Primers used in qPCR analysis.

ID(*)	Gene Product Name		Primers (5' - 3')	Amplicon (bp)	Efficiency (%)	Concentration (nM)
TERG_02214	zinc carboxypeptidase	FW:	ACGAAGGCGTCGTCACTACT	121	101.89	800
		REV:	AAAGCAACAAAGGCAAGAGC			
TERG_07861	subtilase	FW:	AACTATGACCGCTGGGATTG	138	101.72	700
		REV:	CTTCTCCTGGAGGACGTTGA			
TERG_06053	catalase (peroxisomal catalase)	FW:	CTGAATCGCAACGTGAAAGA	118	95.42	700
		REV:	CAAACATACGCGCTTGAAGA			
TERG_08557	serine carboxypeptidase	FW:	TGGACGGAGGCAAGTTTTAC	114	100.03	700
		REV:	TGCAGGTGCTGGTGTCTAAG			
TERG_02979	Delta(24(24(1)))-sterol reductase (Erg4)	FW:	CCTTCCCTTGGTTTTACTTCG	126	102.07	600
		REV:	TGTAGGGAACAGCCTTCTCG			
TERG_03102	sterol 24-C-methyltransferase (Erg6)	FW:	GGCTGACAAAGGGGACATAA	98	101.35	800
		REV:	TGGTCACTCGAAGAGCACTG			
TERG_01252	catalase (catalase A)	FW:	CCACTGGTGATGCAGTCAAT	107	100.45	800
		REV:	CACACCATAGCTCTCCACGA			
TERG_01281	malate synthase	FW:	CGCTGAGGGAAAGAGAGTTG	107	102.92	600
		REV:	CAGCGAGCTGGTACTTGTTG			
TERG_00825	isocitrate lyase	FW:	TATCCATCGTCTGGGAGAGC	107	108.63	700
		REV:	GCTTAGCGTATGCCTTGGAA			
TERG_01076	aconitase	FW:	GGAATGCTTCCCCTTACCTT	121	91.35	800
		REV:	GGTGGACACGGAGAGTCATT			
TERG_01871	acetyl-CoA acetyltransferase	FW:	CAGCAAGTTCACCACTGCTC	92	100.55	600
		REV:	TGATCTCAAAGGCGTCAATG			
TERG_03762	malate dehydrogenase	FW:	GTTGACAGCCCCCTCTACAA	116	96.77	700
		REV:	TGCTCGTACTCGGAGACCTT			
TERG_04125	citrate synthase	FW:	GCTACGTGTCTCAGCGTGAA	107	106.77	600
		REV:	GTGTTCGGTCAAGACACCAG			

TERG_02198	CamK protein kinase	FW:	CAAGGAATCCCAGAGCAAAG	112	95.14	700
		REV:	GTAGAATTCGCACCGAAAGC			
TERG_06573	calcineurin regulatory subunit B	FW:	AGATGGCGGTGGTGACGTA	61	97.80	400
		REV:	CGAACTGAAGGCCGATAACC			
TERG_06392	calmodulin	FW:	GGCCAGAACCCTTCGGAAT	58	107.50	400
		REV:	TGTCGGCGTCAACCTCATT			
TERG_08613	Multidrug resistance protein Mdr2	FW:	TGACGAGGCTACATCAGCAC	104	93.01	800
		REV:	GTCGGTGAGCAACAGCAATA			
TERG_07801	ABC multidrug transporter Mdr4	FW:	GGAAATTGAGCTTCGAGACG	113	94.20	800
		REV:	TTCCAACGATAGCAGTGTGC			
TERG_05742	DNA-dependent RNA polymerase II (rpb2)	FW:	TGCAGGAGCTGGTGGAAGA	59	94.99	300
		REV:	GCTGGGAGGTACTGTTTGATCAA			

(*) TERG: gene accession number at the *T. rubrum* genome database at the Broad Institute.
